# *relA* Inactivation Converts Sulfonamides Into Bactericidal Compounds

**DOI:** 10.3389/fmicb.2021.698468

**Published:** 2021-09-27

**Authors:** Lizhen Si, Jing Gu, Mi Wen, Ruiqi Wang, Joy Fleming, Jinyue Li, Jintian Xu, Lijun Bi, Jiaoyu Deng

**Affiliations:** ^1^Key Laboratory of Special Pathogens and Biosafety, Wuhan Institute of Virology, Chinese Academy of Sciences, Wuhan, China; ^2^University of Chinese Academy of Sciences, Beijing, China; ^3^Key Laboratory of RNA Biology and National Laboratory of Biomacromolecules, CAS Center for Excellence in Biomacromolecules, Institute of Biophysics, Chinese Academy of Sciences, Beijing, China; ^4^School of Stomatology and Medicine, Foshan University, Foshan, China; ^5^Guangdong Province Key Laboratory of TB Systems Biology and Translational Medicine, Foshan, China

**Keywords:** sulfonamides, RelA, reactive oxygen species, ferrous ion, DNA double-strand breaks, bactericidal effects

## Abstract

Folates are required for the *de novo* biosynthesis of purines, thymine, methionine, glycine, and pantothenic acid, key metabolites that bacterial cells cannot survive without. Sulfonamides, which inhibit bacterial folate biosynthesis and are generally considered as bacteriostats, have been extensively used as broad-spectrum antimicrobials for decades. Here we show that, deleting *relA* in *Escherichia coli* and other bacterial species converted sulfamethoxazole from a bacteriostat into a bactericide. Not as previously assumed, the bactericidal effect of SMX was not caused by thymine deficiency. When *E. coli* ∆*relA* was treated with SMX, reactive oxygen species and ferrous ion accumulated inside the bacterial cells, which caused extensive DNA double-strand breaks without the involvement of incomplete base excision repair. In addition, sulfamethoxazole showed bactericidal effect against *E. coli* O157 ∆*relA* in mice, suggesting the possibility of designing new potentiators for sulfonamides targeting RelA. Thus, our study uncovered the previously unknown bactericidal effects of sulfonamides, which advances our understanding of their mechanisms of action, and will facilitate the designing of new potentiators for them.

## Introduction

Folate species are one-carbon units involved in the biosynthesis of purines, thymidine, glycine, methionine, and pantothenic acid in both prokaryotes and eukaryotes (Cossins, 2000; Kompis et al., 2005). Although the cellular requirement for folates is universal, prokaryotes and eukaryotes obtain them *via* different methods. While most microbes are unable to obtain folates from the external environment and must synthesize them *de novo*, mammals can only obtain folate from their diet (Henderson and Huennekens, 1986). The dichotomy of this essential biosynthetic pathway in humans and microbial pathogens makes it an attractive drug target (Bermingham and Derrick, 2002).

Sulfonamides, i.e., compounds targeting the enzyme dihydropteroate synthase (DHPS) that is involved in the bacterial folate biosynthesis pathway, in common use since the 1930s, were the first widely used synthetic antimicrobial agents to treat and control numerous bacterial and parasitic infections (Gaudilliere, 2009). As sulfonamides are generally considered to be bacteriostatic drugs (Seydel, 1968), trimethoprim (TMP), which serves as a potentiator, was later approved for clinical use to enhance the efficacy of sulfonamides and reduce the emergence of resistance (Grunberg and DeLorenzo, 1966). To date, SXT [a fixed combined dosage of TMP and sulfamethoxazole (SMX)] remains one of the key antimicrobial agents recommended by the World Health Organization. However, a report showed that sulfonamides also exert bactericidal effects on *Escherichia coli* when bacterial cells are cultured in minimum medium supplemented with casamino acids and purines (Then and Angehrn, 1973). Thus, after being extensively utilized for several decades, it remains unclear whether sulfonamides are bactericidal. Moreover, resistance to TMP has already emerged (Murray et al., 1982; Toulouse et al., 2020; Manna et al., 2021; Schnetterle et al., 2021), thus, novel potentiators are urgently required to improve the efficacy of SMX and expand its clinical use.

In many bacteria, nutrient starvation, including that of amino acids and fatty acids, stimulates the stringent response, whose hallmark is the accumulation of guanosine 3′,5′-bispyrophosphate (ppGpp), an alarmone and global regulator involved in bacterial stringent response (Chatterji and Ojha, 2001). In gammaproteobacteria, the steady state of ppGpp is maintained by two enzymes, RelA and SpoT. RelA is a synthase, while SpoT is a hydrolase that also exerts low synthase activity (Atkinson et al., 2011). However, most bacteria, including *Mycobacterium tuberculosis*, exhibit a single bifunctional Rel protein as the major RelA/SpoT homolog (Yi and Kim, 2018). ppGpp profoundly affects cellular processes and is also involved in adaptive antibiotic tolerance, or persistence (Abranches et al., 2009; Honsa et al., 2017; Dutta et al., 2019) typically observed toward bactericides rather than bacteriostats (Brauner et al., 2016). Although sulfonamides are known to block bacterial folate biosynthesis, leading to amino acid starvation in bacterial cells, the impact of ppGpp on the antimicrobial efficacy of sulfonamides has not been probed. Thus, it is imperative to determine if ppGpp affects the antimicrobial efficacy of sulfonamides, particularly the bactericidal effects of sulfonamides.

In this study, we measured the effect of sulfonamides on the *relA* mutant strains *in vitro* and *in vivo*. We found that deleting *relA* allowed SMX to exert its bactericidal effect on multiple bacterial species, including *E. coli*, *Salmonella enterica*, and *Mycobacterium tuberculosis*. Meanwhile, other sulfonamides, namely sulfamethazine, sulfadoxin, and sulfisoxazole, could also effectively kill the *E. coli* Δ*relA* mutant. Furthermore, SMX also shows a killing effect on *E. coli* O157 ∆*relA in vivo*. Our results show that the increased accumulation of endogenous reactive oxygen species (ROS) and ferrous ion play crucial roles in the bactericidal effect of SMX, resulting in extensive DNA double-strand breaks (DSBs).

## Materials and Methods

### Bacterial Strains and Plasmids

The information of *E. coli* K-12 W3110, *E. coli* K-12 BW25113, *Salmonella enterica* serovar Typhimurium (*S. enterica*), and *M. tuberculosis* H37Ra strains were listed in Supplementary Table S3. All gene knockout mutant strains of *E. coli* and *S. enterica* were constructed using the λ Red Recombination System as described previously (Datsenko and Wanner, 2000; Baba et al., 2006). The construction of mycobacterial mutant was described below. All plasmids, strains, and primers that we used were listed in Supplementary Tables S3–S6.

### Construction of Mycobacterial Mutant

A modified strategy for specialized transduction was used to construct the *M. tuberculosis* H37Ra Δ*relA* mutant according to a previous publication (Bardarov et al., 2002). Genomic regions flanking *relA*, 820bp upstream (region containing MRA_2611) and 827bp downstream (region containing MRA_2613 and MRA_2614), were amplified by PCR. The primers used for amplification of the upstream of *relA* were *relA*-LFP and *relA*-LRP and those for the region downstream were *relA*-RFP and *relA*-RRP. The recombinant plasmid p0004s-L+R was constructed by inserting the Van91I-digested PCR products into the plasmid p0004s digested with Van91I. Then, the p0004s-L+R was digested with PacI and ligated to the PacI-digested shuttle phasmid vector phAE159. After ligation, the recombinant cosmid phAE159-p0004s-L+R was transducted into *E. coli* HB101 in an *in vitro* λ-packaging reaction (Epicentre: MaxPlax Lambda Packaging Extracts). The phasmid DNA prepared from confirmed selected hygromycin-resistant transductants was electroporated into *M. smegmatis* mc^2^155 to generate the specialized transducing phage. The transducing phage at the most efficient titer was used to infect H37Ra at multiplicity of infection of 10. Successful specialized transduction of H37Ra was confirmed by comparing the size of the PCR-amplified product of hygromycin-resistant colonies with wild-type H37Ra using primers *relA*-LYZ and *relA*-RYZ. The primers we used here were listed in the Supplementary Table S4 and S5.

### Viability Assays

Overnight cultures of *E. coli* and *S. enterica* were diluted 100-fold in fresh Luria-Bertani (LB) medium, grown to an OD_600_=0.6, collected by centrifugation, and washed twice with E minimal medium (MgSO_4_•7H_2_O (0.2g/L), Citric•H_2_O (2g/L), K_2_HPO_4_•3H_2_O (13.09g/L), NaNH_4_HPO_4_•4H_2_O (3.5g/L); Vogel and Bonner, 1956). Cells [10^6^ colony forming units (CFUs)/ml] were incubated in 10ml E minimal medium with 0.5% D-(+)-glucose and the appropriate concentration of SMX (appropriate multiples of MIC) at 37°C for 6 or 8days.

*Mycobacterium tuberculosis* was cultured in 7H9 liquid medium supplemented with 10% (v/v) oleic acid-albumin-dextrose-catalase (OADC), 0.5% (v/v) glycerol and 0.05% (v/v) Tween 80, to log phase (OD_600_~0.8). Cells were washed twice in fresh medium, diluted to OD_600_=0.1 in the same medium, and then treated with 300μg/ml SMX.

Viable cell number was assessed through colony-formation assays (Kohanski et al., 2007). Hundred microliters cultures were serially diluted in E minimal medium (*E. coli*) or 7H9 (*M. tuberculosis*). Ten microliters of each dilution was plated on LB (*E. coli*) or 7H10 (*M. tuberculosis*) agar plates, and the plates were incubated at 37°C overnight (*E. coli*) or for 4weeks (*M. tuberculosis*). CFU/ml values were calculated using the formula: [(colonies)*(dilution factor)]/(volume plated in ml). The limit of detection is 100CFU/ml.

### Drug Susceptibility Testing

*E. coli* and *S. enterica* cells were grown in LB medium to mid-log phase (OD_600_=0.6–0.8) and washed twice with E minimal medium. Resuspended cells were then serially diluted to 10^5^CFU/ml in fresh E minimal medium. Ten microliters dilutions were plated on E minimal medium solid plates (1% agar) containing various concentrations of antibacterial agents. These plates were incubated for approximately 48h at 37°C. The MIC was defined as the lowest concentration of the compound to inhibit 99% of bacterial growth (measured as CFUs). Three independent replicate assays were performed, three randomly selected colonies being assayed each time. Mycobacterial cells were cultured to OD_600_ of 0.5–1.0 and diluted to approximately 10^5^CFU/ml by 10-fold serial dilutions in fresh 7H9 medium with or without 10% OADC. Bacteria were then plated onto 7H10 agar solid plates containing various concentrations of SMX.

### ROS Measurement

*E. coli* K-12 W3110 and *E. coli* K-12 W3110 Δ*relA* were cultured and treated with SMX as described above (viability assay). Cultures were harvested after 8, 16, and 24h of SMX treatment. After suspension in E medium, cells were incubated with 10μM 2′,7′-dichlorodihydrofluorescein diacetate (DCFH-DA, a fluorescent ROS indicator) for 20min in the dark at 37°C and inverted every 5min. Cells were subsequently washed twice with E medium to remove excess extracellular DCFH-DA. Fluorescence was measured at an excitation wavelength of 488nm and an emission wavelength of 525nm using a multimode microplate reader (Synergy H1 Hybrid; BioTek, United States). Relative fluorescence was normalized to CFUs. All data represent the mean of three independent replicates.

### Internal Ferrous Ion Measurement

The precipitations of cell cultures with or without SMX treatment were collected through 0.2μm membrane, and then stored in a refrigerator at −80°C overnight (~12h). The frozen cells were re-suspended in 200μl freshly prepared and precooled PBS, as well as zirconium beads (the ratio of the diameter of 1.5mm and 0.5mm is 3:1) whose volume was approximately one third of the PBS. The mixtures were crushed in a cell breaker for 1min, then immediately incubated on ice for 5min. The above step was repeated for three times and then samples were centrifuge at 13000*g* for 5min at 4°C. The internal ferrous ion concentration was measured by using the iron assay kit according to the manufacturer’s instructions (MAK025).

### Visualization of DNA DSBs in SMX-Treated *Escherichia coli*

Single cell gel electrophoresis assays are based upon the ability of denatured, cleaved DNA fragments to migrate out of the nucleoid, while undamaged DNA remains confined within the nucleoid (due to their slower migration rates), under the influence of an electric field. DNA damage was assessed by evaluating the DNA comet tail shape and migration pattern (Singh et al., 1999; Solanky and Haydel, 2012; Hieke and Pillai, 2018). Neutral comet assays were performed according to the Trevigen’s CometAssay R protocol (Reagent Kit for CometAssay) with modifications.

Wild-type and ∆*relA* mutant cells were harvested after incubation with SMX for 0, 12, 24, 36, and 48h. Twenty microliters of a 10^7^CFU/ml cell suspension was mixed with 200μl LMAgarose, and 50μl of the mixture was pipetted onto a CometSlide. Slides were placed immediately in the dark (at 4°C) until a clear dried ring appeared at the edge of the agarose area. Slides were then placed in a pre-cooled lysis solution and incubated for 1h or overnight. Following bacterial lysis, slides were electrophoresed at 20V for 20min on ice, after gently immersing them in pre-chilled 1× Neutral Electrophoresis Buffer for 30min. Slides were then placed in a DNA precipitation solution for 30min, followed by 70% ethanol for another 30min at 25°C, and dried at 37°C. Slides were stained with 50μl SYBR for 30min in the dark and then air-dried at 37°C in the dark until totally dry.

Fluorescence was observed by confocal microscopy (Andorra Dragonfly 202) at ×630 magnification. The degree of DNA DSBs was expressed as olive tail moment. The tail moment is a measure of damage that combines the amount of DNA in the tail with the distance of migration. Olive tail moment combines tail moment with the value for the difference in DNA gravity between the head and the tail. Olive tail moment was measured in 80 randomly selected cells using the Comet Analysis software.

### Quantitative Real Time PCR

RNA was extracted using a RNeasy Mini Kit (QIAGEN). cDNA was synthesized using the ReverTra Ace qPCR RT Kit (TOYOBO). qRT-PCR was performed using the Power SYBR Green PCR Master Mix (ABI, 4368708). The expression of the genes of interest was normalized to that of *rrsH*. The primers used in this study were shown in Supplementary Table S6.

### Animal Infections and SMX Treatment

A total of 36 6weeks old female BALB/c mice (Vital River Laboratory Animal Technology Co. Ltd., Beijing) were weighed and divided into six groups. All mice were pretreated with 1g/kg streptomycin by gavage once a day for 3days in order to reduce the natural flora to 10^3^CFU/g faeces or less. Half of the mice were then intragastrically infected with 10^10^CFU of *E. coli* O157 pBAD24, and the other half with *E. coli* O157 Δ*relA* pBAD24. Two days post infection, all mice faeces were assessed to determine successful colonization by the afore-mentioned strains. Meanwhile, two groups of mice (one infected with *E. coli* O157 pBAD24 and the other with *E. coli* O157 Δ*relA* pBAD24) were euthanized by cervical dislocation, and caecum colonization was determined at the beginning of drug administration. The remaining four groups of mice were then treated with 100mg/kg SMX or 0.9% NaCl daily by gavage for 4days. SMX (Sigma-Aldrich/Merck, Germany) was administered in 0.9% NaCl at the concentration of 15mg/ml. On a daily basis during the course of SMX treatment, fecal colonization was determined. After 4days of SMX treatment, mice were killed and caecum samples were collected. Fecal and caecum colonization was determined by plating samples on solid LB medium containing ampicillin for viable counts.

### Quantification and Statistical Analysis

Experiments were performed in three biological replicates and at least three technical repetitions. Standard errors were calculated in Microsoft Excel. Visualization of DNA DSBs was observed by confocal microscopy, and the resulting fluorescent (.ims) images was converted to (.tif) images by ImageJ software. Olive tail moment was measured by Comet Analysis software according to the (.tif) images. Graphics were performed in Origin 2018.

## Results

### SMX Is Bacteriostatic Toward *E. coli*, While Deletion of *glyA*, *purH*, *panB*, or *metF* All Led to Cell Death

As an antifolate, SMX simultaneously blocks the biosynthesis of purines, thymidine, glycine, methionine, and pantothenic acid (Cossins, 2000). PurH, GlyA, MetF, and PanB are key proteins in the biosynthesis pathways of purines, glycine, methionine, and pantothenic acid, respectively. Deleting *purH*, *glyA*, *metF*, or *panB* in *E. coli* caused bacterial cell death, suggesting that bacterial cells cannot survive in the minimal medium without any of these molecules (Supplementary Figure S1A). However, SMX showed bacteriostatic, not bactericidal, effects on wild-type *E. coli* (Supplementary Figure S1B). These contradictory results indicate that the mechanisms of action of sulfonamides require further investigation.

### Deleting *relA* Makes Sulfonamides Bactericidal

**Figure 1:**
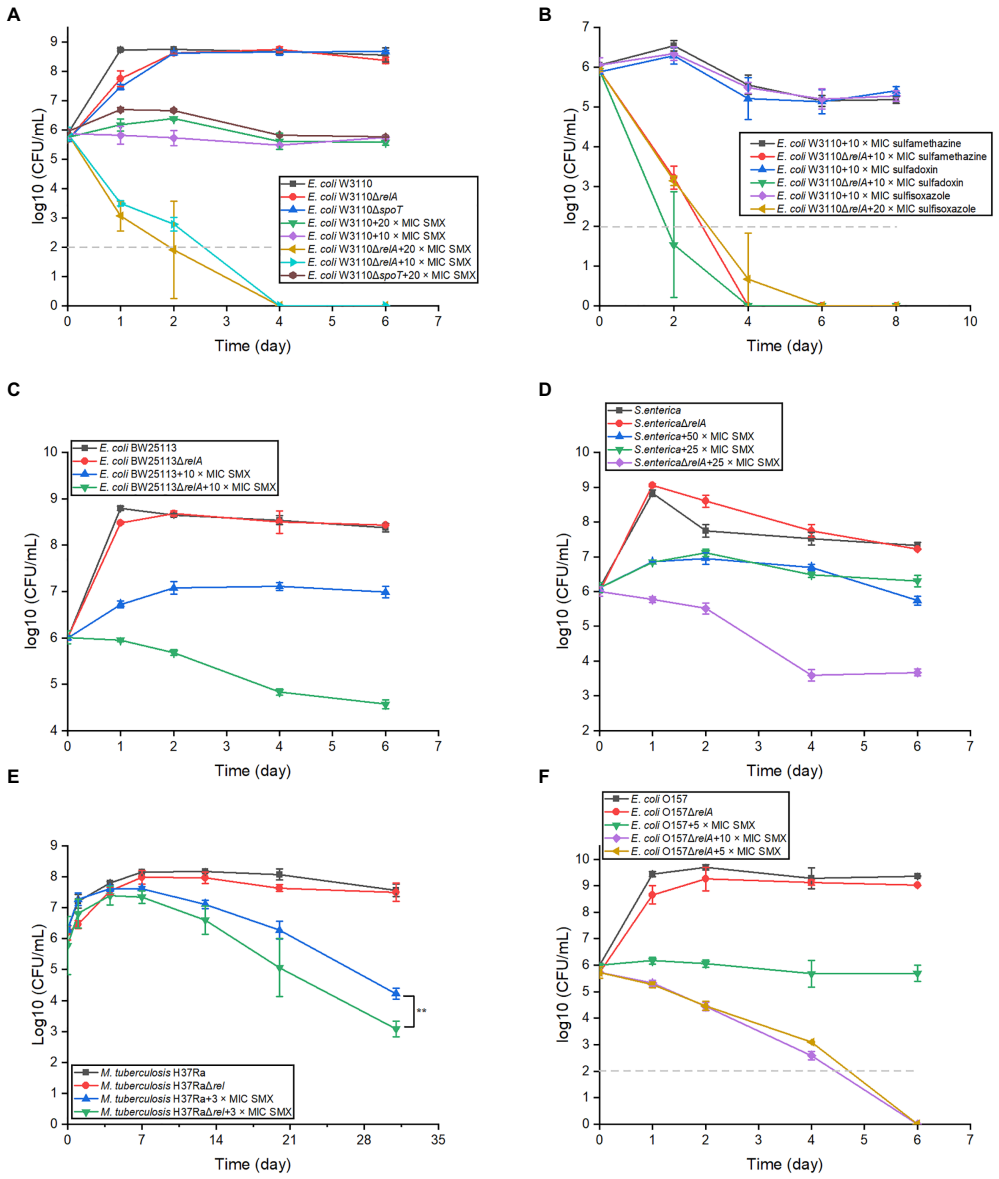
Disrupting the stringent response in different bacterial species converts sulfonamides into bactericides. **(A)** Two key stringent response genes show disparate effects on the bactericidal effect of SMX in *Escherichia coli*. **(B)** Inactivation of *relA* in *E. coli* W3110 also converts other sulfonamides into bactericides, including sulfamethazine (80μg/ml), sulfadoxin (80μg/ml) and sulfisoxazole (40μg/ml). SMX shows bactericidal effect on *E. coli* BW2511 ∆*relA* **(C)**, *S. typhi* ∆*relA* **(D)**, *M. tuberculosis* H37Ra ∆*relA* **(E)**, and pathogenic *E. coli* O157 **(F)**. The MICs of SMX for different bacterial strains were listed in Supplementary Tables S1 and S2. Dashed gray lines represent the limit of detection. In panel **(A–D,F)**, statistical analysis was determined between each ∆*relA* mutant and its equivalent wild type with SMX treatment at each time point, and the differences were all statistically significant (*p*<0.001). Statistical analysis was determined by unpaired two-tailed Student’s t test (^*^p≤0.05, ^**^p≤0.01, *p*>0.05 is not shown). Error bars represent the standard deviation.

To determine if ppGpp has a role in the bactericidal effects of SMX, we first disrupted the stringent response by knocking out *relA* and *spoT* in *E. coli* W3110. Consistent with previous reports, the strain Δ*relA*Δ*spoT* was not able to grow in E minimal medium (Potrykus et al., 2011). When the viability of Δ*relA* and Δ*spoT* single mutants was examined in the presence of SMX, the cell numbers of the Δ*relA* mutant decreased sharply during the first 2days and were subsequently undetectable, while only a slight difference in the Δ*spoT* mutant cell numbers was observed relative to the parental strain (Figure 1A). The killing effect of SMX against the Δ*relA* mutant could be completely reversed by introducing a plasmid bearing an intact copy of *relA* into the mutant (Supplementary Figure S2). We next investigated the viability of the Δ*relA* mutant following exposure to three other sulfonamides [sulfamethazine (80μg/ml), sulfadoxin (80μg/ml), and sulfisoxazole (40μg/ml)], and similar bactericidal effects were observed (Figure 1B). In addition, Δ*relA* mutants of *E. coli* BW25113, *E. coli* O157, *S. enterica*, and *M. tuberculosis* were treated with SMX and showed an approximately two or three log_10_ decrease in viable cell numbers compared with their wild-type strains (Figures 1C–F). Moreover, SMX also showed a killing effect on W3110 Δ*relA* in LB medium (Supplementary Figure S3). These results indicate that RelA, the main ppGpp synthase, impedes the bactericidal effects of sulfonamides.

### Bactericidal Effect of SMX Does Not Depend on Thymineless Death

**Figure 2:**
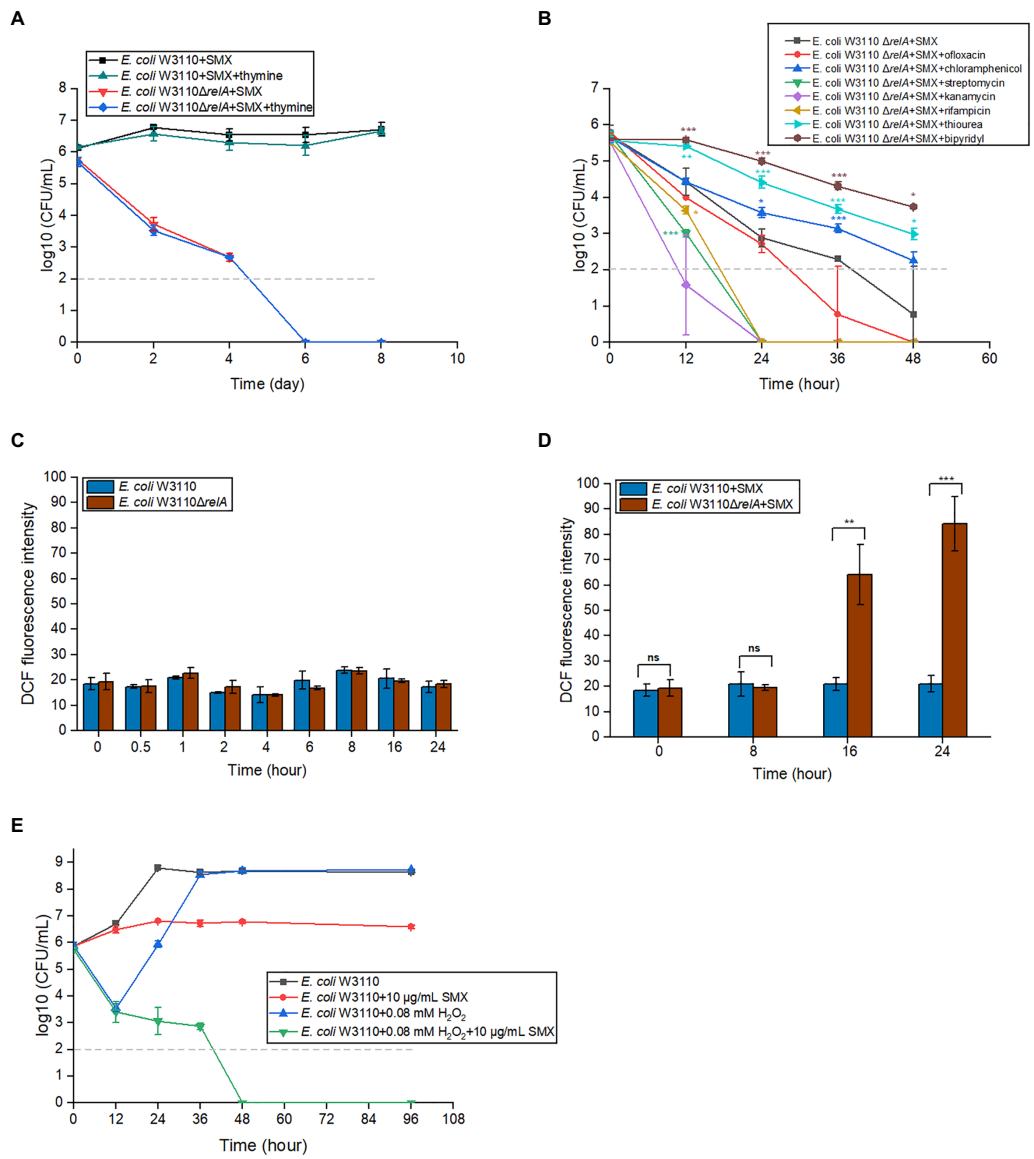
Accumulated ROS, rather than thymine, is responsible for the bactericidal effects of SMX against the Δ*relA* mutant. **(A)** Thymine availability was not related to the killing effect of SMX (10μg/ml) on the wild type or ∆*relA* mutant. The concentration of thymine was 20μg/ml. **(B)** Effects of seven chemical compounds related with ppGpp functions on the killing effect of SMX (10μg/ml). ROS production in the wild type and Δ*relA* mutant without **(C)** or with **(D)** SMX treatment. DCF fluorescence intensity was normalized against bacterial CFUs. **(E)** 0.08mM H_2_O_2_ markedly enhances the killing effect of SMX against *E. coli* W3110. Dashed gray lines represent the limit of detection. Error bars represent the standard deviation. Asterisks indicate statistically significant differences between means as determined by unpaired two-tailed Student’s t test (^*^p≤0.05, ^**^p≤0.01, ^***^p≤0.001; ns, not significant *p*>0.05). In panel A, no statistically significant differences between with/without thymine were detected for either strain at any time point. In panel B, asterisks of the same color as the line indicate the statistical difference between the strain with compound treatment represented by the line and Δ*relA* with SMX treatment, and *p*>0.05 is not shown. In panel C, no statistically significant differences between Δ*relA* mutant and wild type at the same time point. In panel E, statistical analysis was determined between SMX with/without H_2_O_2_, and *p* value of each time point is less than 0.001.

When starved of thymine, cells die rapidly (Barner and Cohen, 1957). This phenomenon, called thymineless death, occurs in both prokaryotes and eukaryotes (Ahmad et al., 1998; Khodursky et al., 2015). Theoretically, SMX blocks the biosynthesis of folate and, hence, thymine. To determine whether the bactericidal effect of SMX on Δ*relA* mutants is also caused by the lack of thymine, we added exogenous thymine to the growth medium along with SMX. The results indicated that exogenous thymine does not influence the bactericidal ability of SMX (Figure 2A).

### 2′-Bipyridy and Thiourea Markedly Protect Δ*relA* Cells From SMX-Induced Killing

ppGpp has been shown to affect DNA replication, RNA transcription, ribosome assembly, and the generation of toxic intermediate ROS (Srivatsan and Wang, 2008; Nguyen et al., 2011; Corrigan et al., 2016). Thus, we tested the effects of inhibitors of these processes on the bactericidal ability of SMX. To this end, we added each inhibitor (0.5×MIC, Supplementary Table S1) together with SMX to the medium of *E. coli* Δ*relA* and measured the mutant viability. The results showed that, in the presence of the iron chelator 2′-bipyridy or the ROS scavenger thiourea, the survival number of *E. coli* Δ*relA* upon SMX treatment was more than 10 times higher than that of the control group (Figure 2B). However, ofloxacin (a DNA gyrase inhibitor) and rifampicin (an RNA polymerase inhibitor) did not show an increase in bacterial survival. Moreover, contradictory results were obtained for different types of protein synthesis inhibitors: though chloramphenicol could partially reduce the killing caused by SMX, streptomycin and kanamycin significantly potentiated it (Figure 2B). These data suggest that the disruption of ppGpp synthesis may induce ROS accumulation, leading to cell death.

### *E. coli* Δ*relA* Accumulates ROS When Treated With SMX

To further verify if the bactericidal effect of SMX on the Δ*relA* mutant is caused by SMX-induced accumulation of ROS, DCFH-DA was used to measure ROS production at different time points in the presence or absence of SMX. Bacterial cells did not accumulate ROS in the absence of SMX (Figure 2C), nor did the wild-type strain in the presence of the drug (Figure 2D). On the contrary, SMX treatment resulted in a significant increase (approximately 3- and 5-fold higher than the ROS production observed in the wild-type strain) in ROS production over time in the Δ*relA* mutant (Figure 2D).

### Exogenous H_2_O_2_ Makes SMX Bactericidal

Next, we wondered if exogenous H_2_O_2_ (an important ROS compound) could also enable SMX to exert a bactericidal effect on *E. coli*. We found that SMX alone showed bacteriostatic effect on *E. coli* W3110 during 96h. When *E. coli* W3110 was treated with 0.08mM hydrogen peroxide alone, the viable cell numbers decreased about three logs during the first 12h. After that, the viable cell numbers increased to the same number that without drug treatment at 36h (Figure 2E). When *E. coli* W3110 was treated with SMX plus hydrogen peroxide, the decrease in cell survival was consistent with that treated with hydrogen peroxide alone during the first 12h and the viable cell numbers kept on decreasing but slowly till 36h. After 48h, the viable cell numbers were below the limit of detection, showing a synergistic effect between these two compounds (Figure 2E).

### Interfering With ROS Production by Blocking the Tricarboxylic Acid (TCA) Cycle, Respiratory Chain, ATP Synthase, or Fe-S Cluster Assembly Protects Δ*relA* From SMX-Induced Killing

ROS, including superoxide anions (O_2_^−^), hydrogen peroxide (H_2_O_2_), and hydroxyl radicals (OH•), are the end products of an oxidative damage pathway and are synthesized by the TCA cycle, respiratory chain, and Fenton reaction (Kohanski et al., 2007). Theoretically, the disruption of these processes should suppress ROS production, and thus reverse the bactericidal effect of SMX. To verify this, we constructed a series of double knockout mutants and evaluated their survival in the presence of SMX (Supplementary Figures S4 and S5).

We constructed 10 double knockout mutants, based on the Δ*relA* strain, in which TCA cycle-related genes were knocked out (Δ*acnA*Δ*relA*, Δ*acnB*Δ*relA*, Δ*sdhA*Δ*relA*, Δ*sdhB*Δ*relA*, Δ*sdhC*Δ*relA*, Δ*sdhD*Δ*relA*, Δ*sucC*Δ*relA*, Δ*sucD*Δ*relA*, Δ*fumE*Δ*relA*, and Δ*mdh*Δ*relA*). As shown in Supplementary Figure S2, Δ*sdhA*Δ*relA*, Δ*sdhB*Δ*relA*, Δ*sdhC*Δ*relA*, Δ*fumE*Δ*relA*, Δ*sdhD*Δ*relA*, and Δ*sucC*Δ*relA* showed a significant increase in survival upon SMX treatment relative to Δ*relA* (Supplementary Figures S4A,B). Inactivation of aconitase-encoding gene *acnA* or *acnB* cannot protect Δ*relA* from killing by SMX (Supplementary Figures S4C,D). Deletion of *mdh*, the gene encoding malate dehydrogenase, could partially reverse the killing effect of SMX, but the protection was significant only at the first 2days of drug treatment (Supplementary Figure S4E).

The *E. coli* aerobic respiratory chain functions with a diverse set of membrane-bound NADH dehydrogenases on the electron input side and three ubiquinol oxidases on the output side (Bekker et al., 2009). We found that the inactivation of cytochrome bo3 ubiquinol oxidase subunits (CyoA, CyoB, and CyoD) in the Δ*relA* mutant did not affect the killing effect of SMX, but deleting *cyoC* in the Δ*relA* mutant could reverse the killing effect of SMX when treated with 10μg/ml of the drug (Supplementary Figure S5A). Considering that the MIC of SMX for Δ*relA*Δ*cyoC* was two times higher than that of Δ*relA*, the viability of Δ*relA*Δ*cyoC* following the treatment with 20μg/ml SMX was also determined, and the reverse effect of Δ*relA*Δ*cyoC* could only be observed at one time point (day 4; Supplementary Figure S5B). On the other hand, the deletion of *cydA* or *cydB* (encoding cytochrome bd-I ubiquinol oxidase) in the Δ*relA* mutant could obviously reverse the killing effect of SMX (Supplementary Figure S5C). Considering that the protection effect of deleting *cydB* is more remarkable than that of deleting *cydA*, cytochrome bd-I ubiquinol oxidase (CydB) is likely the most important terminal oxidase involved in ROS production in the treatment of the Δ*relA* mutant with SMX. In addition, the disruption of NADH dehydrogenases (Ndh, Qor or NuoA) was only able to increase the viability of the Δ*relA* mutant at the fourth day of SMX treatment (Supplementary Figure S5C).

Given that ATP synthase synthesizes most of the cellular ATP by utilizing the electrochemical proton gradient formed through the respiratory chain (Nakanishi-Matsui et al., 2016), we likewise examined the viability of the Δ*atpC*Δ*relA* mutant when treated with SMX. Δ*atpC*Δ*relA* exhibited markedly improved survival compared with Δ*relA* (Supplementary Figure S5D). In addition, deleting *grxD*, which affects the assembly of the Fe-S cluster and iron homeostasis (Wang et al., 2012), totally reversed the killing effect of SMX on Δ*relA* (Supplementary Figure S5D), demonstrating that disrupting the assembly of Fe-S cluster can reverse the bactericidal effect of SMX.

Furthermore, we measured ROS production in these mutants upon SMX treatment. Fluorescence intensity showed that ROS accumulation in the double knockout mutants Δ*sdhA*Δ*relA*, Δ*cydB*Δ*relA*, Δ*grxD*Δ*relA*, and Δ*atpC*Δ*relA* following SMX treatment was greatly reduced compared with Δ*relA* (Supplementary Figure S6). Our data thus suggest that disruption of the TCA cycle, respiratory chain, and Fe-S cluster may interfere with the production of ROS, thereby protecting Δ*relA* from killing by SMX.

### Deletion of *relA* Leads to Increased Expression of Genes Involved in Fe-S Cluster Biogenesis and Ferrous Ion Accumulation Upon SMX Treatment

**Figure 3:**
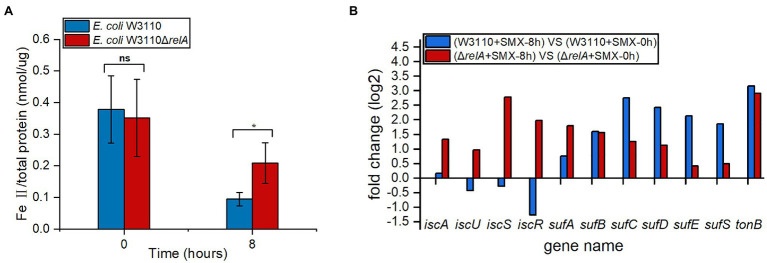
Impacts of RelA on ferrous ion accumulation upon SMX treatment. **(A)** Internal levels of ferrous iron in Δ*relA* and the wild-type strain upon SMX treatment. Asterisks indicate statistically significant differences between means as determined by unpaired two-tailed Student’s t test (^*^p≤0.05, ^**^p≤0.01, ^***^p≤0.001; ns, not significant *p*>0.05). Error bars represent the standard deviation. **(B)** Expression levels of genes associated with iron transport and Fe-S cluster biogenesis.

Since ferrous ion is also required for the bactericidal effect of SMX against Δ*relA*, we compared the accumulation of ferrous ion upon SMX treatment between the wild-type strain and Δ*relA*. The results show that, the amount of ferrous ion accumulated in Δ*relA* was two times higher than that of the wild-type strain upon SMX treatment (Figure 3A). Meanwhile, the data of comparative transcriptional analysis show that, SMX treatment caused increased expression of both the *iscAUSR* and *sufABCDES* genes (two systems for Fe-S cluster biosynthesis in *E. coli*) in Δ*relA*, whereas the same treatment caused increased expression of the *sufABCDES* genes and decreased expression of *iscUSR* in the wild-type strain (Figure 3B). Thus, it SMX treatment leads to increased biogenesis of Fe-S cluster and ferrous ion accumulation in Δ*relA*.

### Incomplete BER Does Not Contribute to the Killing Effect of SMX

ROS are extremely toxic and can readily damage DNA, membrane lipids, and proteins, thereby causing cell death (Dizdaroglu et al., 2002; Imlay, 2003). To date, DNA damage is the best studied form of ROS-related cell damage. Reports in the literature indicate that ROS can damage DNA in different ways and that multiple DNA repair systems, including BER, nucleotide excision repair, and homologous recombination repair, address different types of DNA damage (Jena, 2012). Evaluating the influence of different DNA repair systems on the bactericidal effect of SMX may help in finding clues as to how ROS accumulation induced by the drug results in bacterial death.

Bases and deoxyribose residues in DNA and free nucleotides in the nucleotide pool are susceptible ROS targets, with guanine being the most susceptible due to its low redox potential (Neeley and Essigmann, 2006). Its oxidized base 8-oxo-deoxyguanosine (8-oxo-dG), the most frequent sign of oxidative DNA damage, can be removed by BER, carried out by the MutT, MutM, and MutY DNA glycosylases. Incomplete BER of 8-oxo-dG is much more problematic than the original damage and contributes to antibiotic-induced lethality (Foti et al., 2012; Dwyer et al., 2014; Giroux et al., 2017; ter Kuile and Hoeksema, 2018). Since SMX inhibits folate biosynthesis, which in turn blocks the biosynthesis of essential nucleotides, including dGTP, we reasoned that it is likely that SMX treatment leads to incomplete BER. If this is the case, further disrupting the BER system would reverse the killing effect of SMX against the Δ*relA* mutant. Results showed that SMX only exerted a bacteriostatic effect on the single knockout mutants Δ*mutT*, Δ*mutM*, and Δ*mutY*, similarly to the parental strain (Supplementary Figure S7A). And further deleting Δ*mutT*, Δ*mutM*, or Δ*mutY* could not reverse the killing effect of SMX (Supplementary Figure S7B). On the contrary, further deletion of *mutM*, or Δ*mutT* in Δ*relA* enhanced the killing effect of SMX (Supplementary Figure S7B). These data suggest that BER affect the killing effect of SMX, but incomplete BER does not contribute to the killing effect of SMX.

### Components of the Homologous Recombination Repair System Affect the Bactericidal Effect of SMX, Which Is Directly Caused by DNA DSBs

**Figure 4:**
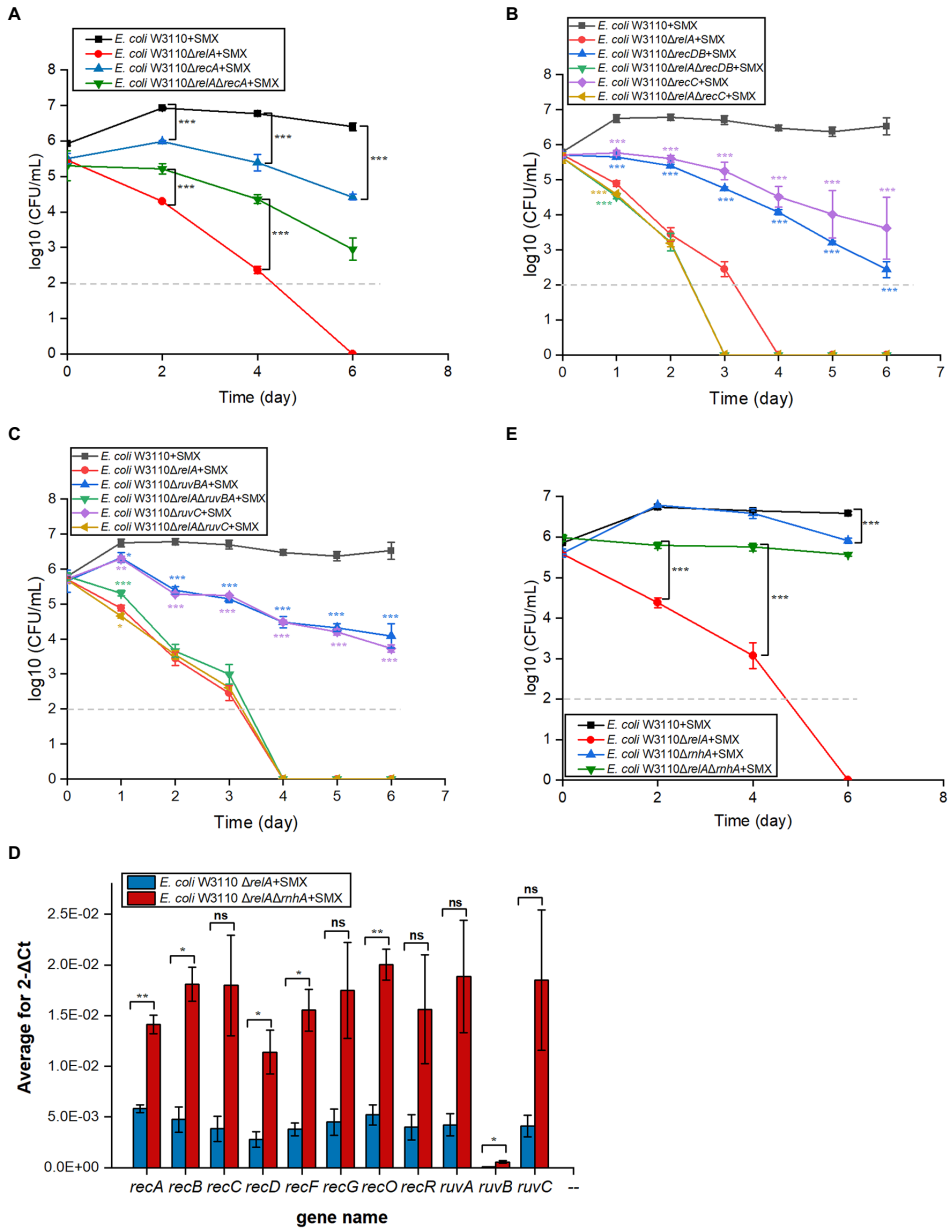
Disruption of the DNA DSB repair system leads to significantly decreased viability in *E. coli* and *E. coli* Δ*relA* upon SMX treatment. **(A–C)** Homologous recombination repair mediates the bactericidal effect of SMX (10μg/ml). **(D)** Expression levels of homologous recombination repair genes in the ∆*relA* and ∆*relA*∆*rnhA* mutants (RT-PCR). **(E)** Deletion of *rnhA* reverses the bactericidal effect of SMX on the ∆*relA* mutant. Dashed gray lines represent the limit of detection. Error bars represent the standard deviation. In panel **(B,C)**, single gene mutant was compared with wild type, while double gene mutant was compared with ∆*relA*; asterisks of the same color as the line indicate the statistical difference between the strain represented by the line and the control with SMX treatment (*p*>0.05 is not shown). Statistical analysis was determined by unpaired two-tailed Student’s *t* test (^*^p≤0.05, ^**^p≤0.01, ^***^p≤0.001; ns, not significant).

RecFOR initiates the repair of DNA single-strand breaks and is coupled with RecA homologous recombination (Cox, 2000; Rocha et al., 2005). We performed viability tests on Δ*recA* and Δ*recFOR* mutants to assess the contribution of single-strand breaks to the bactericidal effect of SMX. While deleting *recA* slightly enhanced the bactericidal effect of SMX, deleting *recF* or *recR* did not (Figure 4A; Supplementary Figure S8A). Similarly, the deletion of *recF*, *recR*, or *recO* in Δ*relA* could not enhance the killing effect of SMX (Supplementary Figure S8A). These results suggest that single-strand breaks are not responsible for the lethality induced by SMX.

**Figure 5:**
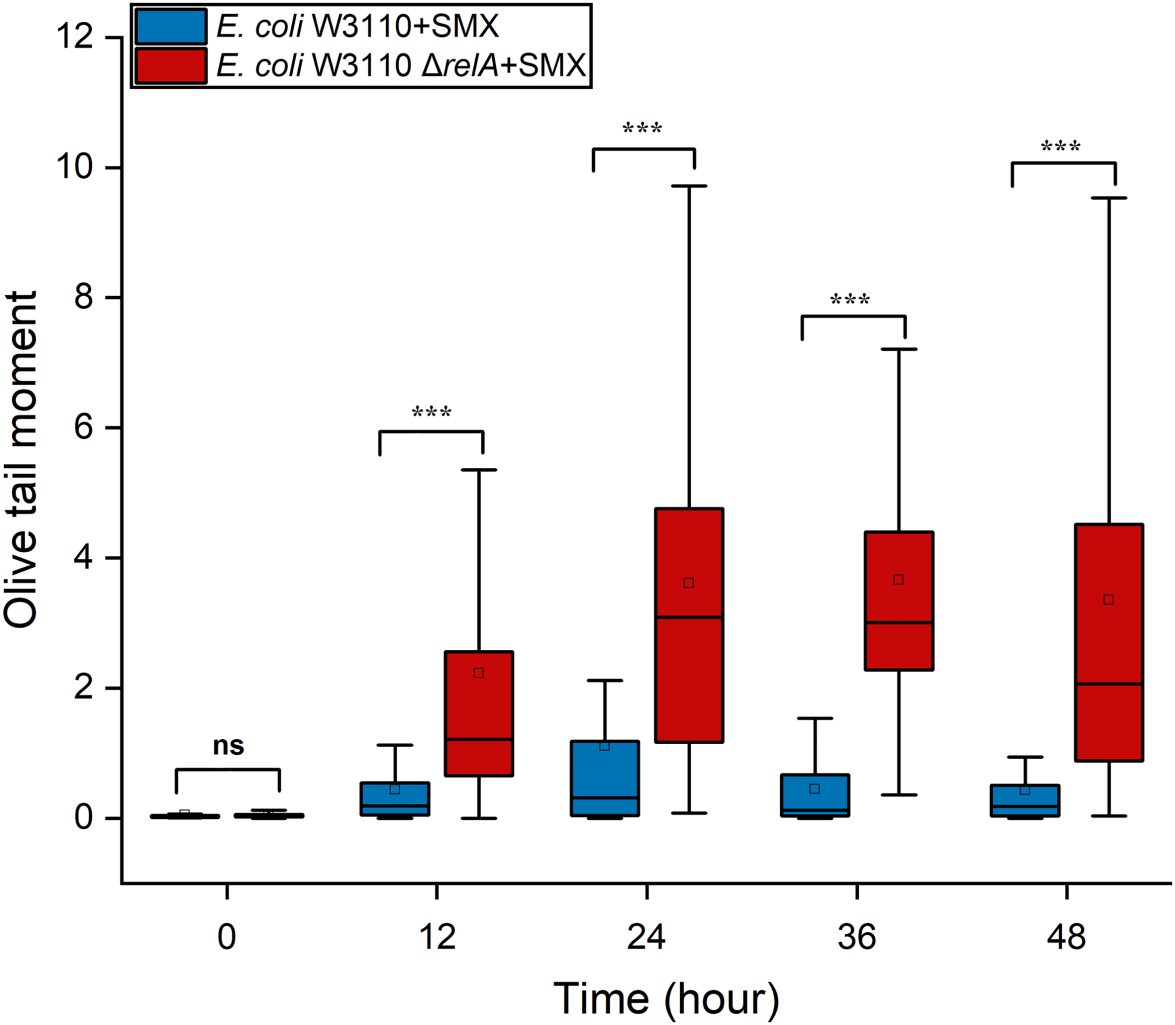
SMX treatment induces extensive DNA DSBs in the Δ*relA* mutant. Box-plot of Olive tail moment levels of DNA from wild-type *E. coli* and the Δ*relA* mutant treated with SMX (10μg/ml). The horizontal line inside the box indicates the median of 80 cells, and the average is shown by the small square above the median line. Asterisks indicate statistically significant differences between means as determined by unpaired two-tailed Student’s *t* test (^*^p≤0.05, ^**^p≤0.01, ^***^p≤0.001; ns, not significant *p*>0.05).

RecBCD initiates the repair of DNA DSBs, recognizes DSB ends, and is also coupled with RecA homologous recombination (Rocha et al., 2005). After the RecA filament polymerizes on the DSB ends, an exchange of homologous pairs occurs between the damaged DNA and its intact sister duplex. The resulting recombination repair intermediates are subsequently resolved by either the RuvABC complex or the RecG helicase (Kuong and Kuzminov, 2010; Azeroglu et al., 2016). Here, we found that the deletion of *recBCD* (Figure 4B) and *ruvABC* (Figure 4C) in wild-type W3110 enabled SMX to exert a killing effect on *E. coli*, whereas that of *recG* did not (Supplementary Figure S8B). These results strongly suggest that DNA DSBs contribute to the bactericidal effect of this drug, since RecA, RecBCD and RuvABC are responsible for the repair of DNA DSBs. Meanwhile, it seems that SMX can induce DNA DSBs in both the wild-type strain and the Δ*relA* mutant. We thus visualized DNA DSBs following SMX treatment using the neutral comet assay (Supplementary Figure S9), and subsequent statistical analysis showed that the olive tail moment of the wild-type strain peaked at 24h, decreasing after 12h and subsequently maintaining its level (Figure 5). The numbers of DNA DSBs were higher in the Δ*relA* mutant than in the wild-type strain (Figure 5). These data suggest that the number of DNA DSBs generated in the wild-type strain upon treatment with SMX did not exceed the repair capacity of the recombinational repair system (Mahaseth and Kuzminov, 2016), while extensive DNA DSBs led to Δ*relA* cell death.

In addition, we tried to induce the expression of genes related to DNA DSB repair in the Δ*relA* mutant by further deleting the RNase HI-encoding gene *rnhA*, which has been shown to be involved in the induction of *recBCD* (Kogoma et al., 1993). As expected, the expression levels of DNA DSB repair genes, such as *recA*, *recB*, *recD*, and *ruvB*, were higher in Δ*rnhA*Δ*relA* than in Δ*relA* (Figure 4D), and the deletion of *rnhA* in the Δ*relA* mutant almost completely reversed the killing effect of SMX, though deleting it in the wild-type strain did not show any effect (Figure 4E). Thus, our data strongly suggest that the homologous recombination system plays an important role in protecting against the lethal effects of SMX and that extensive DNA DSBs caused by SMX are the direct cause of cell death.

### SMX Shows a Bactericidal Effect on *E. coli* O157 Δ*relA* in Mice

**Figure 6:**
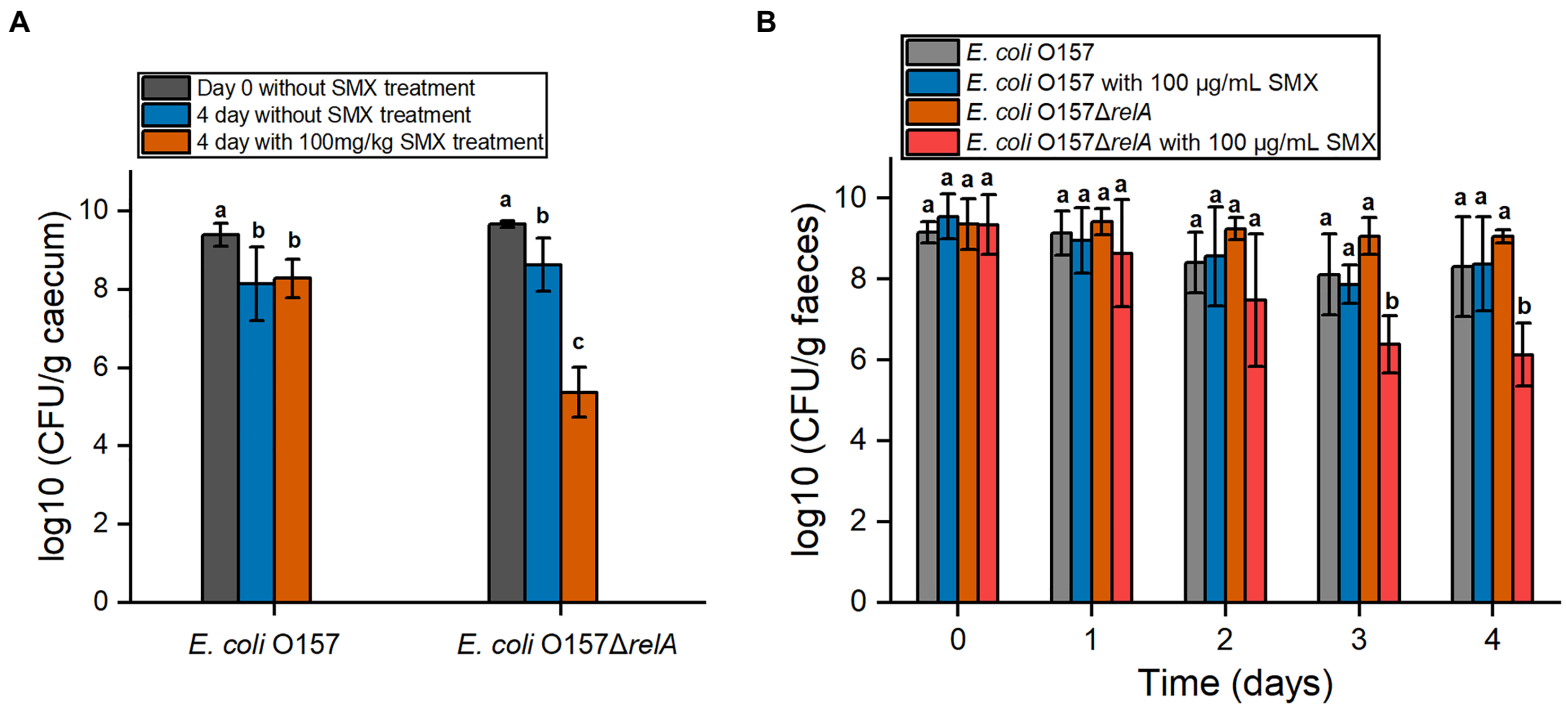
*In vivo* efficacy of SMX against *E. coli* O157 and *E. coli* O157Δ*relA*. **(A)** Viable bacterial cell counts in cecal samples. The day begins from the time of intragastrical administration. **(B)** Viable bacterial cell counts in fecal samples. Letters above bars indicate statistically significant differences between means, as determined by one-way analysis of variance (ANOVA) followed by Tukey’s multiple comparison tests among time points for each strain **(A)** or among strains at each time point **(B)**. Error bars represent the standard deviation.

To further verify whether *relA* gene deletion would affect the bactericidal effect of SMX *in vivo*, the efficacy of SMX against *E. coli* O157 Δ*relA* was also determined in mice. Two days post infection, the number of both O157 and O157Δ*relA* in the caecum as well as the faeces increased to about 10^9^CFU/g, suggesting the success of colonization (Figures 6A,B). After 4days of SMX treatment (100mg/kg), a bactericidal effect of the drug was observed on *E. coli* O157Δ*relA*: viable bacterial counts decreased to about 10^5^CFU/g in the caecum and 10^6^CFU/g in the faeces, which were nearly three logs less than those of the untreated control groups (Figures 6A,B). However, no such bactericidal effect of SMX could be observed on the wild-type strain.

## Discussion

As broad-spectrum antimicrobials, sulfonamides have been extensively utilized clinically for more than half a century. However, it remains obscure whether these old antimicrobial agents are bactericidal. Sulfonamides are generally considered bacteriostatic drugs (Then and Angehrn, 1973); accordingly, we did not observe a bactericidal effect of SMX on *E. coli* W3110. Nevertheless, we also found that bacterial cells could not survive when the synthesis of purines, thymidine, glycine, methionine, or pantothenate was blocked. These contradicting results indicate that the mechanisms of action of sulfonamides require further investigation.

Usually, nutrient starvation (including amino acid starvation) in bacteria induces stringent response mediated by ppGpp. Therefore, we speculated that stringent response might impede the bactericidal effects of sulfonamides. As expected, disrupting the stringent response allowed SMX and different types of sulfonamides to exert their bactericidal effect on three different bacterial species and *E. coli*, respectively. We need to mention that, although SMX exhibits killing activity against Δ*relA*, it is very slow, which does not meet the usual definition of bactericidal (Pankey and Sabath, 2004; Balouiri et al., 2016). So, in this study, we used “bactericidal” to mean that SMX kills bacteria, rather than just stopping them from growing like bacteriostat. Thus, these results demonstrate that stringent response indeed impedes the bactericidal effects of sulfonamides.

Years ago, research showed that cells die rapidly when thymine is absent (Barner and Cohen, 1957). It was also considered that the conversion of multiple nutrient deficiencies caused by SMX to single thymine deficiency could lead to cell death (Cohen and Barner, 1956). Accordingly, the killing effect of SMX was observed when bacterial cells were treated with this drug in minimal medium supplemented with casamino acids and purine, and thus bacterial death occurring under these circumstances was considered to be thymineless death (Then and Angehrn, 1973; Then, 1980; Amyes, 1982). When thymine is deficient, uracil is incorporated into newly replicated DNA. Afterwards, a futile cycle of uracil removal by uracil DNA glycosylase (Ung) and repair of the resulting gaps results in the incorporation of more uracil, eventually leading to fragmented DNA and cell death. Thymineless death can thus be rescued by *ung* deletion or addition of exogenous thymine (Khodursky et al., 2015). Here, however, the addition of exogenous thymine had no impact on the killing effect of SMX on the Δ*relA* mutant, showing that the bactericidal effect of SMX does not depend on thymineless death.

Previously, it was shown that ppGpp can affect antibiotic tolerance by modulating multiple physiological processes (Srivatsan and Wang, 2008; Nguyen et al., 2011; Corrigan et al., 2016); thus, interfering with these processes using their corresponding inhibitors may also diminish the bactericidal effect of SMX on *E. coli* Δ*relA*. Indeed, 2′-bipyridy and thiourea markedly reversed the bactericidal effect of SMX. 2’-Bipyridy can block Fenton reaction-mediated ROS formation by sequestering unbound iron (Imlay et al., 1988), while thiourea can mitigate the effects of ROS damage in both eukaryotes and prokaryotes (Repine et al., 1981; Takahashi et al., 2005). Thus, we speculated that SMX may induce accumulation of ROS in Δ*relA* mutant cells and thus cause cell death. We verified this by measuring ROS production upon SMX treatment and further disrupting the endogenous ROS generation process. Interestingly, we found that SMX also exerted bactericidal effects on *E. coli* W3110 in the presence of exogenous H_2_O_2_, indicating a potential synergistic effect of these two compounds. The contradictory results observed upon the use of different protein synthesis inhibitors might be explained by their capacity to form free radical. Although chloramphenicol, kanamycin and streptomycin are all ribosome inhibitors, the first compound is bacteriostatic agent, while the last two compounds are bactericidal agents. Bactericidal agents can accelerate basal respiratory and lead to produce deleterious ROS, while bacteriostatic agents decelerate respiratory (Dwyer et al., 2014; Lobritz et al., 2015). Interactions between aminoglycosides (like kanamycin and streptomycin) and the ribosome result in protein mistranslation, causing phosphorylation of CpxA and further activation of ArcA, which in turn provokes free radical formation and culminates in cell death (Kohanski et al., 2008). Though bactericidal drugs rather than bacteriostats were generally considered to produce ROS (Kohanski et al., 2007), our results showed that the well-known bacteriostat SMX can also induce ROS accumulation when the bacterial stringent response is disrupted.

Generally, endogenous ROS are generated through aerobic respiration (Imlay and Fridovich, 1991). As the main terminal oxidases of the respiratory chain, CydA and CydB are important contributors to the production of endogenous ROS. Therefore, it was expected that the deletion of *cydA/B* could protect *E. coli* Δ*relA* from killing by SMX. Cytochrome bd-I ubiquinol oxidase catalyzes the two-electron oxidation of ubiquinol and the four-electron reduction of oxygen to water. Since ubiquinol production is mainly dependent on the catalytic synthesis of succinate dehydrogenase (SdhA, SdhB, SdhC, and SdhD) in the TCA cycle, it was not surprising to observe that the deletion of succinate dehydrogenase-encoding genes in the Δ*relA* mutant also greatly reduced the killing effect of SMX. Interestingly, the inactivation of succinyl-CoA synthetase (SucC) also partially reversed the killing effect of SMX, possibly due to insufficient substrate supply for succinate dehydrogenase. Moreover, further deletion of *mdh*, the gene encoding malate dehydrogenase, which catalyzes NADH production in the TCA cycle, also partially reversed the killing effect of SMX. However, further deletion of the aconitase-encoding gene *acnA/B* did not affect the killing effect of SMX on the Δ*relA* mutant. The deletion of *acnB* has previously been shown to quench ROS production and thus reverse the bactericidal effects of multiple antibiotics, probably by affecting bacterial NADH production (Kohanski et al., 2007). Conversely, the inactivation of aconitase has also been shown to cause the accumulation of citrate (Noster et al., 2019), which can promote the production of ROS (Gutteridge, 1990; van de Wier et al., 2013). These two opposite effects may have cancelled each other out when bacterial cells were treated with SMX. Previous research showed that ppGpp mediated suppression of central metabolism could reduce ROS production in *Vibrio cholera*, thereby increase antibiotic tolerance (Kim et al., 2018). Our data indicated that, when bacterial ppGpp synthesis was blocked, the TCA cycle, respiration and energy consumption in bacterial cell might accelerate upon SMX treatment, thus causing the accumulation of ROS.

Interestingly, we found that further deletion of *fumE*, the gene encoding a putative fumarase that contains the Fe-S cluster (Flint et al., 1993; Tseng et al., 2001), almost completely protected Δ*relA* from killing by SMX. Since *E. coli* harbors several copies of fumarase (FumA/B/C/D/E), it is likely that FumE serves as a ferrous iron donor. We also found that the deletion of GrxD, involved in Fe-S assembly, in the Δ*relA* mutant markedly reversed the killing effect of SMX. Furthermore, we found that upon SMX treatment, Δ*relA* accumulated two times more ferrous ion than that of the wild-type strain. These data in combination with that of 2′-bipyridy strongly suggest that ferrous ion is also required for the bactericidal effect of SMX.

ROS accumulation can cause various types of DNA damage, activating elaborate DNA damage repair systems. The disruption of different DNA damage repair systems can thus be used to provide insight into the type of DNA damage that ROS induce upon SMX treatment. When testing the BER system, we found that the deletion of *mutM* or *mutT* enhanced the killing effect of SMX. This suggests that while incomplete BER is not the cause of cell death, un-removed oxidized bases in DNA impact the killing effect of SMX. Moreover, a discrepancy between different components of NER was observed possibly due to the different roles played by the three *E. coli* BER genes (Lu et al., 2001; Dwyer et al., 2009): MutT phosphatase removes 8-oxo-dG from the nucleotide pool through hydrolyzing 8-oxo-dG triphosphate to 8-oxo-dG monophosphate in order to prevent its incorporation into DNA during replication; MutM glycosylase recognizes and excises 8-oxo-dG from DNA when paired with C, G, or T but not with A; MutY glycosylase removes adenine nucleotides paired with 8-oxo-dG. The 8-oxo-G adduct provides a locus for further attack by ROS and reactive nitrogen species, yielding a serial of DNA hyper-oxidation products. This may possibly explain why further deletion of *mutT* or *mutM* in the Δ*relA* strain could enhance the killing effect of SMX. However, disrupting the BER system could not reverse the killing effect of SMX.

In addition to oxidizing free nucleotides, bases, and deoxyribose residues in DNA, ROS can directly generate DNA DSBs. Fe (II) bound to DNA reacts with hydrogen peroxide, producing hydroxyl radicals that preferentially target the sugar-phosphate backbone of DNA. If another Fenton reaction occurs with the same iron atom, another hydroxyl radical is generated nearby, creating a DNA DSB (Mahaseth and Kuzminov, 2016). When examining the homologous recombination repair system, which is responsible for repairing DNA single-strand or DSBs, we found that RecFOR did not exert a significant impact on the killing effect of SMX against wild type, but RecBCD and RuvABC markedly affected it. Subsequently, we compared the number of DNA DSBs between the wild-type strain and the Δ*relA* mutant upon SMX treatment. Results clearly showed that SMX treatment generated more DNA DSBs in the Δ*relA* mutant than that in wild type. In addition, we activated the homologous recombination repair system by inactivating its known repressor RhnA, which almost completely reversed the killing effect of SMX on the Δ*relA* mutant. Altogether, these data strongly suggest that SMX-induced accumulated ROS generate DNA DSBs, which is the direct cause of bacterial cell death. Based on a previously published observation, in which damaged bases in DNA were found to impede nonhomologous end joining (a crucial pathway involved in DNA DSB repair; Datta et al., 2011), we speculate that un-removed oxidized bases in DNA might also impede the homologous recombination process, possibly explaining why the disruption of the BER system significantly enhances the killing effect of SMX.

Taken together, our findings show that disrupting the stringent response in different bacterial species, including *E. coli*, enables the bactericidal effect of well-known bacteriostat sulfonamides. Unlike the bactericidal effect of the other antifolate TMP, that of SMX does not rely on thymineless death. So far, the bactericidal effects of many commonly used antibiotics have been shown to be dependent on ROS through the Fenton reaction. This is the first report showing that the bactericidal effects of the well-known bacteriostat sulfonamides can also induce the accumulation of both ROS and ferrous ion, which induces DNA DSBs without incomplete BER involvement. However, the mechanism of accumulation of ROS and ferrous ion upon SMX treatment in stringent response mutant strain requires further investigation. What’s more, as SMX also exerts bactericidal effect on *E. coli* O157 Δ*relA in vivo*, and the ppGpp-mediated stringent response only occurs in prokaryotes, it is plausible that the design of new inhibitors of ppGpp synthases, such as RelA, will yield novel potentiators of sulfonamides. This study deepens our understanding of the mechanisms of action of sulfonamides and will facilitate the design of new potentiators of these compounds.

## Data Availability Statement

The original contributions presented in the study are included in the article/Supplementary Material, further inquiries can be directed to the corresponding author.

## Ethics Statement

The animal study was reviewed and approved by Institutional Review Board, Wuhan Institute of Virology, Chinese Academy of Sciences.

## Author Contributions

LS and JD designed the studies, wrote the manuscript, performed the data analysis, and acquired high resolution figures. LS carried out the experiments in the assistance of JG, MW, JL, RW, and JX. JF and LB revised the manuscript. All authors contributed to the article and approved the submitted version.

## Funding

This work was supported by the Strategic Priority Research Program of the Chinese Academy of Sciences (grant no. XDB29020000).

## Conflict of Interest

The authors declare that the research was conducted in the absence of any commercial or financial relationships that could be construed as a potential conflict of interest.

## Publisher’s Note

All claims expressed in this article are solely those of the authors and do not necessarily represent those of their affiliated organizations, or those of the publisher, the editors and the reviewers. Any product that may be evaluated in this article, or claim that may be made by its manufacturer, is not guaranteed or endorsed by the publisher.
