# Supplementary material for: relA Inactivation Converts Sulfonamides Into Bactericidal Compounds
Source: Front Microbiol. 2021 Sep 27;12:698468. doi: 10.3389/fmicb.2021.698468 (PMC8503649; doi:10.3389/fmicb.2021.698468)
Supplement: Supplementary file 1 [file Data_Sheet_1.docx]

Supplementary Material





**Supplementary Figure 1.** **SMX-blocking of folate synthesis shows only bacteriostatic effects against *E. coli*, while deleting *purH*, *glyA*, *metF* or *panB* leads to cell death.** (A) Survival of ∆*purH*, ∆*glyA*, ∆*metF* and ∆*panB* in E minimal medium with 0.5% glucose. Dashed gray line represents the limit of detection. (B) The effect of SMX (10 μg/mL) on *E. coli* W3110. Error bars represent the standard deviation.


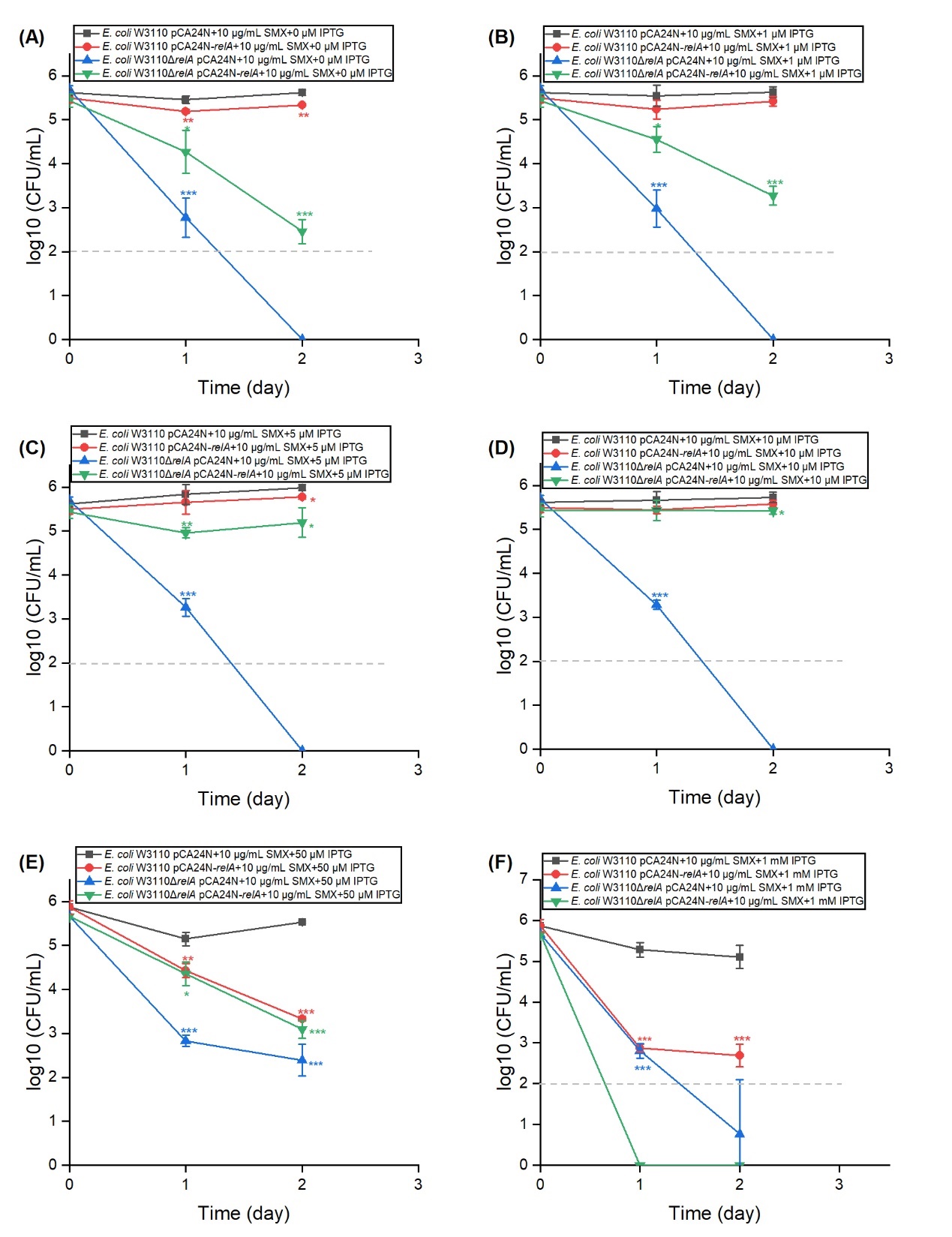


**Supplementary Figure 2. Complementing the *E. coli* W3110 Δ*relA* mutant by using the pCA24N::*relA* recombinant plasmid.** To carefully modulate the expression level of RelA, different concentrations of IPTG were used: 0 μM (A), 1 μM (B), 5 μM (C), 10 μM (D), 50 μM (E), and 1 mM (F). SMX and IPTG were added into the growth medium at the same time. Dashed gray lines represent the limit of detection. The asterisks of the same color as the line indicate the statistical difference between the strain represented by the line and wild type. Statistical analysis was determined by unpaired two-tailed Student’s t test (**P* ≤ 0.05, ***P* ≤ 0.01, ****P* ≤ 0.001, and *P*>0.05 is not shown). Error bars represent the standard deviation.





**Supplementary Figure 3. Effects of SMX on *E. coli* W3110 and the Δ*relA* mutant in LB medium.** The concentration of SMX is 4 folds of MIC in LB medium. Dashed gray lines represent the limit of detection. Statistical analysis was determined between ∆*relA* mutant and wild type at each time point by unpaired two-tailed Student’s t test, and the differences were all statistically significant (*P* <0.001). Error bars represent the standard deviation.

**

**

**Supplementary Figure 4**. **Interfering with ROS production by blocking the TCA cycle protects Δ*relA* from SMX-induced killing.** (A–E) Log change in CFU/mL of different mutant strains related to TCA cycle genes following exposure to SMX (10 μg/mL). Deletion of succinate dehydrogenase (A), deletion of fumarase or succinyl-CoA synthetase (B), deletion of aconitase (C and D), deletion of malate dehydrogenase (E). Each deletion indicated above was introduced in both wild-type *E. coli* W3110 and *E. coli* W3110Δ*relA*. The MIC values of these strains were listed in Supplementary Table 2. For comparison, survival of the wild type and Δ*relA* treated with SMX are also shown. Dashed gray lines represent the limit of detection. Error bars represent the standard deviation. The asterisks of the same color as the line indicate the statistical difference between the mutant strain represented by the line and Δ*relA*. Statistical analysis was determined by unpaired two-tailed Student’s t test (**P* ≤ 0.05, ***P* ≤ 0.01, ****P* ≤ 0.001, and *P*>0.05 is not shown).

**

**

**Supplementary Figure 5.** **Interfering with ROS production by blocking the** **respiratory chain or Fe-S cluster assembly protects Δ*relA* from SMX-induced killing.** (A–C) Log change in CFU/mL of respiratory chain associated mutant strains following exposure to SMX. Deletion of cytochrome bo3 ubiquinol oxidase deficiency (A and B), deletion of NADH dehydrogenase (*nuoA*, *qor*, and *ndh* mutants) or cytochrome bd-I ubiquinol oxidase (*cydA* and *cydB* mutants) (C), and each mutation indicated above was introduced in both wild-type *E. coli* W3110 and *E. coli* W3110Δ*relA*. (D) The effect of blocking the synthesis of ATP or GrxD (involved in iron metabolism) on the viability of ∆*relA*. For comparison, survival of the wild type and Δ*relA* treated with SMX are also shown. The concentration of SMX used in (A), (C), and (D) was 10 μg/mL, while that in (B) was 20 μg/mL (based on the different MIC for these strains (see also Supplementary Table 2)). Error bars represent the standard deviation. Dashed gray lines represent the limit of detection. The asterisks of the same color as the line indicate the statistical difference between the mutant strain represented by the line and Δ*relA*. Statistical analysis was determined by unpaired two-tailed Student’s t test (**P* ≤ 0.05, ***P* ≤ 0.01, ****P* ≤ 0.001, and *P*>0.05 is not shown).





**Supplementary Figure 6**. **SMX-induced ROS accumulation is significantly reduced by disrupting the TCA cycle, respiratory chain, ATP synthase, or Fe-S cluster assembly.** Bacterial cells were treated with 10 μg/mL SMX for 24 h. DCF fluorescence intensity was normalized to bacterial CFUs. Error bars represent the standard deviation. Letters above bars indicate statistically significant differences between means, as determined by one-way analysis of variance (ANOVA) followed by Tukey’s multiple comparison test.

**

**

**Supplementary Figure 7.** **BER components affect the killing effect of SMX.** The effect of deletion of BER components (*mutY*, *mutM*, *mutT*) on the viability of wild type (A) and ∆*relA* mutant (B) following exposure to SMX. The concentration of SMX used in these experiments was 10 μg/mL. Error bars represent the standard deviation. The asterisks of the same color as the line indicate the statistical difference between the mutant strain represented by the line and wild type (in panel A) or Δ*relA* (in panel B). Statistical analysis was determined by unpaired two-tailed Student’s t test (**P* ≤ 0.05, ***P* ≤ 0.01, ****P* ≤ 0.001, and *P*>0.05 is not shown).





**Supplementary Figure 8. The influences of SSR components and recG on the killing effect of SMX.** (A) The effect of the deletion of SSB repair related gene (*recF*, *recO*, *recR*) on the viability of the ∆*relA* mutant following exposure to SMX. (B) The effect of deletion of *recG* on the viability of the ∆*relA* mutant following exposure to SMX. For comparison, survival of the wild type and Δ*relA* as well as single gene mutant strains of those components, treated with SMX are also shown. The concentration of SMX used in these experiments was 10 μg/mL. Error bars represent standard deviation. Dashed gray lines represent the limit of detection. The asterisks of the same color as the line indicate the statistical difference between the mutant strain represented by the line and Δ*relA*. Statistical analysis was determined by unpaired two-tailed Student’s t test (**P* ≤ 0.05, ***P* ≤ 0.01, ****P* ≤ 0.001, and *P*>0.05 is not shown).

**
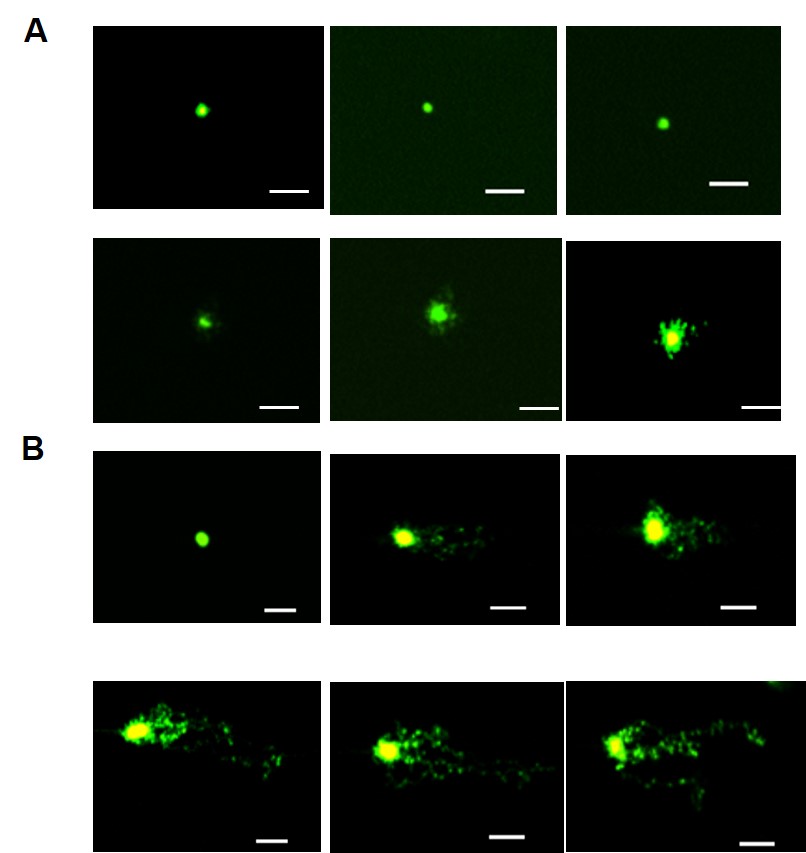
**

**Supplementary Figure 9. Fluorescence images obtained from neutral comet assays.** The DNA shape of neutral comet assay for wild-type (A) and ∆*relA* mutant (B) cells are shown. The length of the comet tails represents the extent of lesions after SMX treatment (10 μg/mL). Scale bar = 10 μm.

**Supplementary Table 1. MIC of various antimicrobial agents for *Escherichia coli* W3110 and the ∆*relA* mutant in E minimal medium.**

| **Antimicrobial agents** | **MIC** | |
| --- | --- | --- |
|  | ***E. coli* W3110** | ***E. coli* W3110 Δ*relA*** |
| Ofloxacin | 0.04 μg/mL | 0.04 μg/mL |
| Chloramphenicol | 2.5 μg/mL | 2.5 μg/mL |
| Streptomycin | 8 μg/mL | 4 μg/mL |
| Kanamycin | 10 μg/mL | 5 μg/mL |
| Rifampicin | 4 μg/mL | 2 μg/mL |
| Thiourea | 0.2 M | 0.1 M |
| Bipyridyl | 0.5 mM | 0.5 mM |
| Sulfamethazine | 8 μg/mL | 8 μg/mL |
| Sulfadoxin | 8 μg/mL | 8 μg/mL |
| Sulfisoxazole | 4 μg/mL | 2 μg/mL |

MIC, minimum inhibitory concentration.

**Supplementary Table 2. MIC of SMX for some bacterial strains used in this study in E minimal medium.**

| **Strain** | **SMX MIC (μg/mL)** |
| --- | --- |
| *Escherichia coli* W3110 | 1.00 |
| *E. coli* W3110Δ*relA* | 0.50 |
| *E. coli* W3110Δ*spoT* | 1.00 |
| *E. coli* W3110Δ*relA*Δ*sdhA* | 1.00 |
| *E. coli* W3110Δ*relA*Δ*sdhB* | 0.25 |
| *E. coli* W3110Δ*relA*Δ*sdhC* | 0.50 |
| *E. coli* W3110Δ*relA*Δ*sdhD* | 0.50 |
| *E. coli* W3110Δ*relA*Δ*sucC* | 1.00 |
| *E. coli* W3110Δ*relA*Δ*sucD* | 1.00 |
| *E. coli* W3110Δ*relA*Δ*fumE* | 1.00 |
| *E. coli* W3110Δ*relA*Δ*mdh* | 1.00 |
| *E. coli* W3110Δ*relA*Δ*acnA* | 1.00 |
| *E. coli* W3110Δ*relA*Δ*acnB* | 1.00 |
| *E. coli* W3110Δ*relA*Δ*cyoA* | 1.00 |
| *E. coli* W3110Δ*relA*Δ*cyoB* | 1.00 |
| *E. coli* W3110Δ*relA*Δ*cyoC* | 2.00 |
| *E. coli* W3110Δ*relA*Δ*cyoD* | 1.00 |
| *E. coli* W3110Δ*relA*Δ*cydA* | 1.00 |
| *E. coli* W3110Δ*relA*Δ*cydB* | 1.00 |
| *E. coli* W3110Δ*relA*Δ*atpC* | 0.25 |
| *E. coli* W3110Δ*relA*Δ*nuoA* | 1.00 |
| *E. coli* W3110Δ*relA*Δ*ndh* | 1.00 |
| *E. coli* W3110Δ*relA*Δ*qor* | 1.00 |
| *E. coli* W3110Δ*relA*Δ*grxD* | 1.00 |
| *E. coli* W3110Δ*relA*Δ*rnhA* | 1.00 |
| *E. coli* BW25113 | 1.00 |
| *E. coli* BW25113Δ*relA* | 1.00 |
| *Salmonella enterica* | 4.00 |
| *S. enterica* Δ*relA* | 2.00 |
| *Mycobacterium tuberculosis H37Ra* | 100.00 |
| *M. tuberculosis H37Ra* Δ*relA* | 100.00 |
| *E. coli* O157 | 2.00 |
| *E. coli* O157Δ*relA* | 1.00 |

SMX, sulfamethoxazole; MIC, minimum inhibitory concentration.

**Supplementary Table 3. List of bacterial strains and plasmids used in this study.**

| **Strain and plasmid** | **Relevant characteristic** | **Source** |
| --- | --- | --- |
| *E. coli* W3110 | Wild-type | (Li and Zhang, 2007) |
| *E. coli* W3110 Δ*relA* | *relA*::FRT^1^ | This study |
| *E. coli* W3110 Δ*spoT* | *spoT*::FRT | This study |
| *E. coli* W3110 Δ*panB* | *panB*::FRT | This study |
| *E. coli* W3110 Δ*purH* | *purH*::FRT | This study |
| *E. coli* W3110 Δ*metF* | *metF*::FRT | This study |
| *E. coli* W3110 Δ*glyA* | *glyA*::FRT | This study |
| *E. coli* W3110 Δ*recA* | *recA*::FRT | This study |
| *E. coli* W3110 Δ*rnhA* | *rnhA*::FRT | This study |
| *E. coli* W3110 Δ*recF* | *recF*::FRT | This study |
| *E. coli* W3110 Δ*recO* | *recO*::FRT | This study |
| *E. coli* W3110 Δ*recG* | *recG*::FRT | This study |
| *E. coli* W3110 Δ*recR* | *recR*::FRT | This study |
| *E. coli* W3110 Δ*recC* | *recC*::FRT | This study |
| *E. coli* W3110 Δ*recDB* | *recDB*::FRT | This study |
| *E. coli* W3110 Δ*ruvC* | *ruvC*::FRT | This study |
| *E. coli* W3110 Δ*ruvBA* | *ruvBA*::FRT | This study |
| *E. coli* W3110 Δ*mutM* | *mutM*::FRT | This study |
| *E. coli* W3110 Δ*mutY* | *mutY*::FRT | This study |
| *E. coli* W3110 Δ*mutT* | *mutT*::FRT | This study |
| *E. coli* W3110 Δ*relA*Δ*sdhA* | *relA*::FRT *sdhA*::FRT | This study |
| *E. coli* W3110 Δ*relA*Δ*sdhB* | *relA*::FRT *sdhB*::FRT | This study |
| *E. coli* W3110 Δ*relA*Δ*sdhC* | *relA*::FRT *sdhC*::FRT | This study |
| *E. coli* W3110 Δ*relA*Δ*sdhD* | *relA*::FRT *sdhD*::FRT | This study |
| *E. coli* W3110 Δ*relA*Δ*sucC* | *relA*::FRT *sucC*::FRT | This study |
| *E. coli* W3110 Δ*relA*Δ*sucD* | *relA*::FRT *sucD*::FRT | This study |
| *E. coli* W3110 Δ*relA*Δ*fumE* | *relA*::FRT *fumE*::FRT | This study |
| *E. coli* W3110 Δ*relA*Δ*acnA* | *relA*::FRT *acnA*::FRT | This study |
| *E. coli* W3110 Δ*relA*Δ*acnB* | *relA*::FRT *acnB*::FRT | This study |
| *E. coli* W3110 Δ*relA*Δ*atpC* | *relA*::FRT *atpC*::FRT | This study |
| *E. coli* W3110 Δ*relA*Δ*mdh* | *relA*::FRT *mdh*::FRT | This study |
| *E. coli* W3110 Δ*relA*Δ*recA* | *relA*::FRT *recA*::FRT | This study |
| *E. coli* W3110 Δ*relA*Δ*recG* | *relA*::FRT *recG*::FRT | This study |
| *E. coli* W3110 Δ*relA*Δ*recF* | *relA*::FRT *recF*::FRT | This study |
| *E. coli* W3110 Δ*relA*Δ*rnhA* | *relA*::FRT *rnhA*::FRT | This study |
| *E. coli* W3110 Δ*relA*Δ*recO* | *relA*::FRT *recO*::FRT | This study |
| *E. coli* W3110 Δ*relA*Δ*recR* | *relA*::FRT *recR*::FRT | This study |
| *E. coli* W3110 Δ*relA*Δ*recC* | *relA*::FRT *recC*::FRT | This study |
| *E. coli* W3110 Δ*relA*Δ*recDB* | *relA*::FRT *recDB*::FRT | This study |
| *E. coli* W3110 Δ*relA*Δ*ruvC* | *relA*::FRT *ruvC*::FRT | This study |
| *E. coli* W3110 Δ*relA*Δ*ruvBA* | *relA*::FRT *ruvBA*::FRT | This study |
| *E. coli* W3110 Δ*relA*Δ*mutM* | *relA*::FRT *mutM*::FRT | This study |
| *E. coli* W3110 Δ*relA*Δ*mutY* | *relA*::FRT *mutY*::FRT | This study |
| *E. coli* W3110 Δ*relA*Δ*mutT* | *relA*::FRT *mutT*::FRT | This study |
| *E. coli* W3110 Δ*relA*Δ*cyoA* | *relA*::FRT *cyoA*::FRT | This study |
| *E. coli* W3110 Δ*relA*Δ*cyoB* | *relA*::FRT *cyoB*::FRT | This study |
| *E. coli* W3110 Δ*relA*Δ*cyoC* | *relA*::FRT *cyoC*::FRT | This study |
| *E. coli* W3110 Δ*relA*Δ*cyoD* | *relA*::FRT *cyoD*::FRT | This study |
| *E. coli* W3110 Δ*relA*Δ*nuoA* | *relA*::FRT *nuoA*::FRT | This study |
| *E. coli* W3110 Δ*relA*Δ*ndh* | *relA*::FRT *ndh*::FRT | This study |
| *E. coli* W3110 Δ*relA*Δ*qor* | *relA*::FRT *qor*::FRT | This study |
| *E. coli* W3110 Δ*relA*Δ*cydA* | *relA*::FRT *cydA*::FRT | This study |
| *E. coli* W3110 Δ*relA*Δ*cydB* | *relA*::FRT *cydB*::FRT | This study |
| *E. coli* W3110 Δ*relA*Δ*spoT* | *relA*::FRT *spoT*::FRT | This study |
| *E. coli* BW25113 | Wild-type | CICC 23872 |
| *E. coli* BW25113 Δ*relA* | *relA*::FRT | This study |
| *S. enterica* | Wild-type | CICC 22956 |
| *S. enterica* Δ*relA* | *relA*::FRT | This study |
| *M. tuberculosis* H37Ra | Wild-type | Ph. D William R Jacobs Jr |
| *M. tuberculosis* H37Ra Δ*rel* | *rel* mutant | This study |
| *E. coli* HB101 | host bacteria for plasmid | Ph. D William R Jacobs Jr |
| *Mycobacterium smegmatis* mc^2^155 | for the construction of mycobacterial mutant | Ph. D William R Jacobs Jr |
| p0004s | homologous arm construction plasmid for *M. tuberculosis* H37Ra | Ph. D William R Jacobs Jr |
| phAE159 | Shuttle plasmid for bacteriophage packaging | Ph. D William R Jacobs Jr |
| *E. coli* O157 | Wild-type | CICC 21530 |
| *E. coli* O157 Δ*relA* | *relA*::FRT | This study |
| *E. coli* O157 pBAD24 | pBAD24, Amp^+^ | This study |
| *E. coli* O157 Δ*relA* pBAD24 | *relA*::FRT; pBAD24, Amp^+^ | This study |
| *E. coli* W3110 pCA24N | pCA24N, Cm^+^ | This study |
| *E. coli* W3110 Δ*relA* pCA24N | pCA24N, Cm^+^ | This study |
| *E. coli* W3110 pCA24N::*relA* | pCA24N bearing the full-length *relA* | This study |
| *E. coli* W3110 Δ*relA* pCA24N::*relA* | pCA24N bearing the full-length *relA* | This study |

^1^ FRT indicates the fragment that was left on elimination of the kanamycin cassette using the FLP recombinase.

**Supplementary Table 4. Primers used in the construction of knockout strains**.

| **Name^1^** | **Primer sequence (5’-3’)** |
| --- | --- |
| acnA-koF | ATGTCGTCAACCCTACGAGAAGCCAGTAAGGACACGTTGCAGGCCAAAGAATATGAATATCCTCCTTAG |
| acnA-koR | TTACTTCAACATATTACGAATGACATAATGCAAAATGCCGTCGTTCTGGTTGTAGGCTGGAGCTGCTTCG |
| acnB-koF | GTGCTAGAAGAATACCGTAAGCACGTAGCTGAGCGTGCCGCTGAGGGGATATATGAATATCCTCCTTAG |
| acnB-koR | TTAAACCGCAGTCTGGAAAATCACCCCATCGGCTTTCTCGGTGTACTGAGTGTAGGCTGGAGCTGCTTCG |
| atpC-koF | ATGGCAATGACTTACCACCTGGACGTCGTCAGCGCAGAGCAACAAATGTTATATGAATATCCTCCTTAG |
| atpC-koR | TTACATCGCTTTTTTGGTCAACTCGATAACGCGCAGCTGCGCGATCGCTTTGTAGGCTGGAGCTGCTTCG |
| cydA-koF | ATGTTAGATATAGTCGAACTGTCGCGCTTACAGTTTGCCTTGACCGCGATATATGAATATCCTCCTTAG |
| cydA-koR | TTAGCGTGCCGGCTGAGTAGTCGTGGAAGACTGCTCAAAGTGATAGCGACTGTAGGCTGGAGCTGCTTCG |
| cydB-koF | ATGATCGATTATGAAGTATTGCGTTTTATCTGGTGGCTGCTGGTTGGCGTATATGAATATCCTCCTTAG |
| cydB-koR | TTAGTACAGAGAGTGGGTGTTACGTTCAATATCTTCTTTGGTGATACGACTGTAGGCTGGAGCTGCTTCG |
| cyoA-koF | ATGAGACTCAGGAAATACAATAAAAGTTTGGGATGGTTGTCATTATTTGCATATGAATATCCTCCTTAG |
| cyoA-koR | TTAATGGGCGGATTCCGCGTGGCTCATGTCCATGCCTTCCATACCTTCGTTGTAGGCTGGAGCTGCTTCG |
| cyoB-koF | ATGTTCGGAAAATTATCACTTGATGCAGTCCCGTTCCATGAACCTATCGTATATGAATATCCTCCTTAG |
| cyoB-koR | TCAGTTGCCATTTTTCAGCCCTGCCTTAGTAATCTCATCGAAATGCTGGTTGTAGGCTGGAGCTGCTTCG |
| cyoC-koF | ATGGCAACTGATACTTTGACGCACGCGACTGCCCACGCGCACGAACACGGATATGAATATCCTCCTTAG |
| cyoC-koR | TTACATCGCCCCCATCAGATAAACAACAGTGAACACACAGATCCAAACCATGTAGGCTGGAGCTGCTTCG |
| cyoD-koF | ATGAGTCATTCTACCGATCACAGCGGCGCGTCCCATGGCAGCGTAAAAACATATGAATATCCTCCTTAG |
| cyoD-koR | TTAGTGCATCATCATGTTGTAGTTGAGGTTCCACATAATCCAGATGGAGCTGTAGGCTGGAGCTGCTTCG |
| fumE-koF | ATGGCGACGCTGACAGAAGATGATGTGCTTGAGCAACTGGATGCACAGGAATATGAATATCCTCCTTAG |
| fumE-koR | TCACGGCGTTAATTCCTTTAAGGTGGTCGTTACTGCCAGCGTCAGCGCCGTGTAGGCTGGAGCTGCTTCG |
| mdh-koF | ATGAAAGTCGCAGTCCTCGGCGCTGCTGGCGGTATTGGCCAGGCGCTTGCATATGAATATCCTCCTTAG |
| mdh-koR | TTACTTATTAACGAACTCTTCGCCCAGGGCGATATCTTTCTTCAGCGTATTGTAGGCTGGAGCTGCTTCG |
| mutM-koF | ATGCCTGAATTACCCGAAGTTGAAACCAGCCGCCGCGGCATAGAACCGCAATATGAATATCCTCCTTAG |
| mutM-koR | TTACTTCTGGCACTGCCGACAATAAAACGTTGCCCGCTGCGCATGTTTAGTGTAGGCTGGAGCTGCTTCG |
| mutY-koF | ATGCAAGCGTCGCAATTTTCAGCCCAGGTTCTGGACTGGTACGATAAATAATATGAATATCCTCCTTAG |
| mutY-koR | CTAAACCGGCGCGCCAGTGCGTAACTGCTGTAACAAACGCTCCACGGGAGTGTAGGCTGGAGCTGCTTCG |
| mutT-koF | ATGAAAAAGCTGCAAATTGCGGTAGGTATTATTCGCAACGAGAACAATGAATATGAATATCCTCCTTAG |
| mutT-koR | CTACAGACGTTTAAGCTTCGCAATTACCGGTTCATTGGCTGGCGGAAAATTGTAGGCTGGAGCTGCTTCG |
| ndh-koF | TTGACTACGCCATTGAAAAAGATTGTGATTGTCGGCGGCGGTGCTGGTGGATATGAATATCCTCCTTAG |
| ndh-koR | TTAATGCAACTTCAAACGCGGACGGATAACGCGGTTAATACTCCCCACCATGTAGGCTGGAGCTGCTTCG |
| nuoA-koF | ATGAGTATGTCAACATCCACTGAAGTCATCGCTCATCACTGGGCATTCGCATATGAATATCCTCCTTAG |
| nuoA-koR | TTAGCGTTGACGATTAGCGATACTGTTCGTTTCCGGGTTCATACGCTCGCTGTAGGCTGGAGCTGCTTCG |
| panB-koF | ATGAAACCGACCACCATCTCCTTACTGCAGAAGTACAAACAGGAAAAAAAATATGAATATCCTCCTTAG |
| panB-koR | TTAATGGAAACTGTGTTCTTCGCCCGGATAAACGCCGGACTCCACTTCAGTGTAGGCTGGAGCTGCTTCG |
| purH-koF | ATGCAACAACGTCGTCCAGTCCGCCGCGCTCTGCTCAGTGTTTCTGACAAATATGAATATCCTCCTTAG |
| purH-koR | TTAATGGCGGAAGTGGCGCATGTCGGTGAAGAGCATCGCAATACCGTGCTTGTAGGCTGGAGCTGCTTCG |
| qor-koF | ATGGCAACACGAATTGAATTTCACAAGCACGGTGGCCCGGAAGTACTTCAATATGAATATCCTCCTTAG |
| qor-koR | TTATGGAATCAGCAGGCTGGAACCTTGCGTCGCCCGGCTTTCCAGAATCTTGTAGGCTGGAGCTGCTTCG |
| recA-koF | ATGGCTATCGACGAAAACAAACAGAAAGCGTTGGCGGCAGCACTGGGCCAATATGAATATCCTCCTTAG |
| recA-koR | TTAAAAATCTTCGTTAGTTTCTGCTACGCCTTCGCTATCATCTACAGAGATGTAGGCTGGAGCTGCTTCG |
| recC-koF | ATGTTAAGGGTCTACCATTCCAATCGTCTGGACGTGCTGGAAGCGTTGATATATGAATATCCTCCTTAG |
| recC-koR | TTAAAGCGAAACAGCGGTAACAGGAAACGTTGCGACTGTTCAACGATGGCTGTAGGCTGGAGCTGCTTCG |
| recDB-koF | ATGAGTGATGTCGCCGAGACACTAGATCCTTTGCGCTTGCCCTTACAGGATATGAATATCCTCCTTAG |
| recDB-koR | TTATTCCCGTGAACTAAACAACGCCGCCAGACCACTGCGCCGCTCAGTATGTAGGCTGGAGCTGCTTCG |
| recF-koF | ATGTCCCTCACCCGCTTGTTGATCCGCGATTTCCGCAACATTGAAACCGCATATGAATATCCTCCTTAG |
| recF-koR | TTAATCCGTTATTTTACCCTTTTCCACGGTAAACATCTTCGAATTTTCGTTGTAGGCTGGAGCTGCTTCG |
| recG-koF | ATGAAAGGTCGCCTGTTAGATGCTGTCCCACTCAGTTCCCTAACGGGCGTATATGAATATCCTCCTTAG |
| recG-koR | TTACGCATTCGAGTAACGTTCCGTCTCCGGCATCCAGCGTTCTATCAGGGTGTAGGCTGGAGCTGCTTCG |
| recO-koF | ATGGAAGGCTGGCAGCGCGCATTTGTCCTGCATAGTCGCCCGTGGAGCGAATATGAATATCCTCCTTAG |
| recO-koR | TCATTCATAATGTGTTTTCACCGTTCGCTTAGGCATAAACTGCCGGAACATGTAGGCTGGAGCTGCTTCG |
| recR-koF | ATGCAAACCAGCCCGCTGTTAACACAGCTTATGGAAGCACTGCGCTGTCTATATGAATATCCTCCTTAG |
| recR-koR | TTAAAAACGAATCTTATGACGCCCGGCAAGGGAGTGTGACAACGTGGTGCTGTAGGCTGGAGCTGCTTCG |
| relA-koF | ATGGTTGCGGTAAGAAGTGCACATATCAATAAGGCTGGTGAATTTGATCCATATGAATATCCTCCTTAG |
| relA-koR | CTAACTCCCGTGCAACCGACGCGCGTCGATAACATCCGGCACCTGGTTGATGTAGGCTGGAGCTGCTTCG |
| rnhA-koF | ATGCTTAAACAGGTAGAAATTTTCACCGATGGTTCGTGTCTGGGCAATCCATATGAATATCCTCCTTAG |
| rnhA-koR | TTAAACTTCAACTTGGTAGCCTGTATCTTCCAGTGTGGGATTCATCGCCGTGTAGGCTGGAGCTGCTTCG |
| ruvBA-koF | GTGATAGGCAGACTCAGAGGCATCATCATTGAAAAACAACCCCCGCTGGTATATGAATATCCTCCTTAG |
| ruvBA-koR | TTACGGCATTTCTGGCGGCGTTATGCCAAAGTGATTCCACGCCCGCGTCGTGTAGGCTGGAGCTGCTTCG |
| ruvC-koF | ATGGCTATTATTCTCGGCATTGATCCGGGTTCGCGCGTGACCGGCTACGGATATGAATATCCTCCTTAG |
| ruvC-koR | TTAACGCAGTCGCCCTCTCGCCAGGTTCAGCCGCGATTCGCTCATCTGCATGTAGGCTGGAGCTGCTTCG |
| sdhA-koF | ATGAAATTGCCAGTCAGAGAATTTGATGCAGTTGTGATTGGTGCCGGTGGATATGAATATCCTCCTTAG |
| sdhA-koR | TTAGTAAGTACGAATCTTCGGCGGGAATGCCGGGCGCAGTTTCGGTTCCATGTAGGCTGGAGCTGCTTCG |
| sdhB-koF | ATGAGACTCGAGTTTTCAATTTATCGCTATAACCCGGATGTTGATGATGCATATGAATATCCTCCTTAG |
| sdhB-koR | TTACGCATTACGTTGCAACAACATCGACTTGATATGGCCGATGGCGCGCGTGTAGGCTGGAGCTGCTTCG |
| sdhC-koF | ATGATAAGAAATGTGAAAAAACAAAGACCTGTTAATCTGGACCTACAGACATATGAATATCCTCCTTAG |
| sdhC-koR | TTACCATACGAGGACTCCTGCGAGAAGTGAAAGCACGACAGTAATAACAATGTAGGCTGGAGCTGCTTCG |
| sdhD-koF | ATGGTAAGCAACGCCTCCGCATTAGGACGCAATGGCGTACATGATTTCATATATGAATATCCTCCTTAG |
| sdhD-koR | TCACACACCCCACACCACAACGAATCCATAAATCACGTAAACCACCAGTGTGTAGGCTGGAGCTGCTTCG |
| spoT-koF | TTGTATCTGTTTGAAAGCCTGAATCAACTGATTCAAACCTACCTGCCGGAATATGAATATCCTCCTTAG |
| spoT-koR | TTAATTTCGGTTTCGGGTGACTTTAATCACGTCTGGCATCACGCGGATTTTGTAGGCTGGAGCTGCTTCG |
| sucC-koF | ATGAACTTACATGAATATCAGGCAAAACAACTTTTTGCCCGCTATGGCTTATATGAATATCCTCCTTAG |
| sucC-koR | TTATTTCCCCTCCACTGCGGCAACAACCTGCTGAGCTGCATCCGTCAGACTGTAGGCTGGAGCTGCTTCG |
| sucD-koF | ATGTCCATTTTAATCGATAAAAACACCAAGGTTATCTGCCAGGGCTTTACATATGAATATCCTCCTTAG |
| sucD-koR | TTATTTCAGAACAGTTTTCAGTGCTTCACCGATATCCGCCAGGCTGCGAATGTAGGCTGGAGCTGCTTCG |
| ST^2^-relA-koF | TCCTGTAACCCGGCGACGCTGGCGCGCGATAGTGAAGCGCTGGTCAATGCATATGAATATCCTCCTTAG |
| ST-relA-koR | TTACCCCCCGTGCAGTCGCCGTGCATCAATCACATCCGGCACCTGGTTCATGTAGGCTGGAGCTGCTTCG |
| BW^3^-relA-koF | ATGGTTGCGGTAAGAAGTGCACATATCAATAAGGCTGGTGAATTTGATCCATATGAATATCCTCCTTAG |
| BW-relA-koR | CTAACTCCCGTGCAACCGACGCGCGTCGATAACATCCGGCACCTGGTTGATGTAGGCTGGAGCTGCTTCG |
| *relA*-LFP^4^ | TTTTTTTTCCATAAATTGGGCGCTGGCGCGGTGACGATGGCCA |
| *relA*-LRP^4^ | TTTTTTTTCCATTTCTTGGGCGGTGCAACCGCTTGCGCCGTGAGCT |
| *relA*-RFP^5^ | TTTTTTTTCCATAGATTGGTACGACGTCTACCG GGTGACCTCG |
| *relA*-RRP^5^ | TTTTTTTTCCATCTTTTGGCGCGGTAGTCGTCACGGTGGTGGT |
| O^6^-relA-koF | ATGGTTGCGGTAAGAAGTGCACATATCAATAAGGCTGGTGAATTTGATCCATATGAATATCCTCCTTAG |
| O^6^-relA-koR | CTAACTCCCGTGCAACCGACGCGCGTCGATAACATCCGGCACCTGGTTGATGTAGGCTGGAGCTGCTTCG |

^1^ F: forward primer, R: reverse primer.

^2^ ST represents *S. enterica* strain.

^3^ BW represents *E. coli* W3110 BW25113 strain.

^4^ The primers are used for amplification of the upstream region of *relA* of *M. tuberculosis* H37Ra.

^5^ The primers are used for amplification of the downstream region of *relA* of *M. tuberculosis* H37Ra.

^6^ O represents *E. coli* O157 strain.

**Supplementary Table 5. Primers used in PCR checking.**

| **Name^1^** | **Sequence** | **Name** | | **Sequence** | |
| --- | --- | --- | --- | --- | --- |
| acnA-iF | AAGAGAATCAGGGCTTCGCA | recDB-iF | TTGATTTACTGCCCGAGAGC | |  |
| acnA-iR | CGACTTCACTCTTTGCGCTG | recDB-iR | AAGGCATGGCTATTCTGTCG | |  |
| acnB-iF | AGGAGCGTGAAGAGAATCGC | recF-iF | CTATAGCGGTGCGGAGATGG | |  |
| acnB-iR | CCCATGGACATACGCACCTT | recF-iR | ATACCATGTGGTGCAGACCG | |  |
| atpC-iF | AGCAGGCGTTCTACATGGTC | recG-iF | GATGAAGCGGCGGTAGAAGT | |  |
| atpC-iR | TCCAGACTGGCTTTTGTGCT | recG-iR | CAAGGCGAGATCGAAGCTGA | |  |
| cydA-iF | ACTCTCGGAGTCTTCATGCG | recO-iF | CAGCAAAAATGTGGCTTCGT | |  |
| cydA-iR | ACATAGAAGCCGGAGAACGC | recO-iR | GTCTCGGTTACGTTGACGAT | |  |
| cydB-iF | CCTGTTCCTGGTGGCAGAAT | recR-iF | TGGCTTTAAGATGCCGTTCT | |  |
| cydB-iR | TCCAGTTCACTGGTCTTCGC | recR-iR | CATCACCTTCGTACAGGTCC | |  |
| cyoA-iF | CCTGCTGGCTACGCATCATA | relA-iF | CATCACCTTCGTACAGGTCC | |  |
| cyoA-iR | ATCCCGTGGAATTGAGGTCG | relA-iR | CATCACCTTCGTACAGGTCC | |  |
| cyoB-iF | AATGCAGTCGCTCATCAGGT | rnhA-iF | GCGCTTGGTTGCTTACAACT | |  |
| cyoB-iR | CCGCCCATTAAAGGGGTTGA | rnhA-iR | CAGCCGATCGGGTTTGAGAT | |  |
| cyoC-iF | CCAGAACGGAATCACCGTCA | ruvB-iF | TTTCAGGAGGCGCAATGTAT | |  |
| cyoC-iR | ACGTGGATTACTACGTGCCG | ruvB-iR | TTTCAGGAGGCGCAATGTAT | |  |
| cyoD-iF | AGATCAGGCCTTTCACCAGC | ruvC-iF | GCTATGTATTCCGGTGGGTG | |  |
| cyoD-iR | TCTGCACGTCACTTCTGGTC | ruvC-iR | GATCCCGTCTACCAAAGCTG | |  |
| fumE-iF | TCCCAGAAGGTGGTTAGGGT | sdhA-iF | ACTACGTTAAACCGCTGGCT | |  |
| fumE-iR | TAACCGTGTGTACTCGCTCG | sdhA-iR | AGCGGGCGAATCACAATCTT | |  |
| mdh-iF | TCTGCTCTGGAGACGATGGA | sdhB-iF | AGAGTCGGAATCCATGACGC | |  |
| mdh-iR | CCACCTGTTGGAATGTTGCG | sdhB-iR | GGCTCGCGAAGGAACCTTTA | |  |
| mutM-iF | TCGATCCAGTTGTTCGCCAG | sdhC-iF | AATCCCGACGTCTCCAGGTA | |  |
| mutM-iR | AGCGTTTATGCCGGATGGTA | sdhC-iR | AGGCGAAGAAACCGATCCAG | |  |
| mutY-iF | GTTAGTTGCCGGATGCAAGC | sdhD-iF | ACGTCGTCGTAGGTATTCGC | |  |
| mutY-iR | AGCCCTCGATATGCACCTCT | sdhD-iR | GAACGGGTCGGGAAGACTTT | |  |
| ndh-iF | CTTATCAGGCCTACCGAACG | spoT-iF | TGATTGCCGCTGTTACGGAT | |  |
| ndh-iR | GGAGTCTGAAAGGATGCCTG | spoT-iR | TGATTGCCGCTGTTACGGAT | |  |
| nuoA-iF | TCCAGAGGGTCGGTTACGAT | sucC-iF | ACAAGCGATGCCTGATGTGA | |  |
| nuoA-iR | TGCTTCACAACGGACACGAT | sucC-iR | CCGCCAACCATTTTAGTGCC | |  |
| panB-iF | CCGAAGGGGCGAAAACTAAATCC | sucD-iF | GTTAACGTACCGGTCGTGGT | |  |
| panB-iR | ATGCCAGACAGCACAACATCAAT | sucD-iR | AACCGAAGAGATGAGCCGTC | |  |
| purH-iF | ACGCACAGAGTTATCCACAATCA | recA-iF | TACGTCGCAGTTCTTGCTCA | |  |
| purH-iR | CGCCGTTACCAATCACTAATACT | recA-iR | TCGTGCTGATTATGCCGTGT | |  |
| qor-iF | AAGTGCCGAAATATCAGCGT | recC-iF | AATGCTTTGAACCAGGTGCT | |  |
| qor-iR | CATCATCCCAGCGTTCGTTA | recC-iR | AGGTAATGTTGGCGATGGTG | |  |
| BW^2^-relA-iF | TACGGGCTCTTTACGCACTG | ST^3^-relA-iF | TCCCTCAGCAGACGAAGAGA | |  |
| BW-relA-iR | CTCGGGATAGCGAAGCGTTA | ST-relA-iR | GTGAAAACGCCATCCGCAAT | |  |
| relA-LYZ^4^ | GTGAGCAGGAATCCGACGCGATG | O^5^-relA-iF | CGCTTTACGCTACTGTGGAT | |  |
| relA-RYZ^4^ | CGTCGCATCTTGACTATCGTCGGT | O^5^-relA-iR | GGATATACCATTGCGCGACT | |  |

^1^ F: forward primer, R: reverse primer.

^2^ ST represents *S. enterica* strain.

^3^ BW represents *E. coli* W3110 BW25113 strain.

^4^ The primers are used to identify whether successful construction of *M. tuberculosis* H37RaΔ*relA*.

^5^ O represents *E. coli* O157 strain.

**Supplementary Table 6. Primers used in qPCR.**

| **Name^1^** | **Sequence (5’-3’)** |
| --- | --- |
| recA-R | CAGCGTGTTGGACTGCTTCA |
| recA-F | CTTGCGGCACGTATGATGAG |
| recB-F | GCGGCGTTATTTATCTGTTCCT |
| recB-R | TCCCCTGTTGCGGATGTT |
| recC-F | GACGATTCCACGTTGCAAAA |
| recC-R | TGTTGCCTTCGTAAGCCTGAA |
| recD-F | TCGCCGTCTGTCGCTGTA |
| recD-R | GGCGATTGCCGCACTTAA |
| ruvA-F | GGTTTGCATGGCGATCTCTT |
| ruvA-R | GCTGGCAGGAGACGTGAGTAC |
| ruvB-F | GATTCAGCAAGGCTTTTTGCA |
| ruvB-R | CCGCGTCGTCGCCATA |
| ruvC-F | CCCACTGCCACGTTAGTCAGA |
| ruvC-R | CAGGTTCAGCCGCGATTC |
| recG-F | CGTTACCCACAACAGGCAAA |
| recG-R | CCGTCTCCGGCATCCA |
| recF-F | GCGATCAGTGCTGAACACGTT |
| recF-R | TTTCCACGGTAAACATCTTCGA |
| recO-F | GGTTTATCGCAAGCGTCGTT |
| recO-R | GGAAATTCCCGTGCGTTTAA |
| recR-F | AACTACATTGCCGAGCTTTGC |
| recR-R | AACGCCATGAGCGATTCG |
| rrsH-F | CGGAGTCGCTAGTAATCGCA |
| rrsH -R | GATCCCACCTTCGACAGCTC |

^1^ F: forward primer, R: reverse primer.

**Supplementary Table 7. List of chemicals and reagent kits used in this study.**

| **Chemicals and Reagent Kits** | **Source** | **Identifier** |
| --- | --- | --- |
| Ofloxacin | Sigma-Aldrich | Cat#82419-36-1 |
| Chloramphenicol | MDBio, Inc | Cat#9004-39-1 |
| Streptomycin | MDBio, Inc | Cat#57-92-1 |
| Kanamycin | MDBio, Inc | Cat#25389-94-0 |
| Rifampicin | Sigma-Aldrich | Cat#13292-46-1 |
| Thiourea | Sinopharm | Cat#62-56-6 |
| Bipyridyl | Sinopharm | Cat#366-18-7 |
| Sulfamethoxazole | Sigma-Aldrich | Cat#723-46-6 |
| Sulfamethazine | Sigma-Aldrich | Cat#57-68-1 |
| Sulfadoxin | Sigma-Aldrich | Cat#5018-54-2 |
| Sulfisoxazole | Sigma-Aldrich | Cat#127-69-5 |
| MgSO_4_ •7H_2_O | aladdin | Cat#10034-99-8 |
| Citric•H_2_O | aladdin | Cat#5949-29-1 |
| K_2_HPO_4_•3H_2_O | aladdin | Cat#16788-57-1 |
| NaNH_4_HPO_4_•4H_2_O | aladdin | Cat#7783-13-3 |
| D-(+)-glucose | Sinopharm | Cat#50-99-7 |
| OADC | BD | Cat#9206278 |
| Glycerol | General-reagent | Cat#56-81-5 |
| Tween 80 | Sigma-Aldrich | Cat#9005-65-6 |
| CometAssay R | Trevigen | Cat#4250-050-K |
| DCFH-DA | Beyotime | Cat#S0033S |
| MaxPlax Lambda Packaging Extracts | Epicentre | Cat#MP5120 |
| RNeasy Mini Kit | QIAGEN | Cat#74106 |
| ReverTra Ace qPCR RT Kit | TOYOBO | Cat# FSQ-101 |
| Iron assay kit | Sigma-Aldrich | Cat# MAK025 |

Reference

Li, Y., and Zhang, Y. (2007). PhoU is a persistence switch involved in persister formation and tolerance to multiple antibiotics and stresses in *Escherichia coli*. *Antimicrobial agents and chemotherapy* 51(6)**,** 2092-2099. doi: 10.1128/AAC.00052-07.
